# Elucidating the roles of microRNA-103a-3p in trophoblast invasion and SOX4-mediated extravillous differentiation induced by activin A

**DOI:** 10.1038/s41419-026-08665-6

**Published:** 2026-04-10

**Authors:** Jiamin Xie, Matthew J. Shannon, Hua Zhu, Minyue Dong, Alexander G. Beristain, Peter C. K. Leung

**Affiliations:** 1 The British Columbia Children’s Hospital Research Institute, Vancouver, BC Canada; 2 Department of Obstetrics and Gynecology, The University of British Columbia, Vancouver, BC Canada; 3 Women’s Hospital, School of Medicine, Zhejiang University, Hangzhou, China; 4 Key Laboratory of Reproductive Genetics (Zhejiang University), Ministry of Education, Hangzhou, China

**Keywords:** Predictive markers, Differentiation, Stem-cell differentiation

## Abstract

The dysregulation of growth factors is associated with defective trophoblast invasion, which leads to uteroplacental malperfusion due to the inadequate remodeling of spiral arteries. Pregnancy disorders, including preeclampsia, particularly early-onset preeclampsia, are closely related to compromised placental function caused by aberrant trophoblast invasion. Activin A, a growth factor detectable in serum that belongs to the transforming growth factor-β (TGF-β) superfamily, has been implicated in the development of preeclampsia, as evidenced by its elevated serum levels and its role in regulating trophoblast invasion. However, the existing research on its regulatory mechanisms in trophoblast invasion has focused mainly on intracellular, nonsecretory epithelial-mesenchymal transition (EMT) markers in conventional trophoblast cell lines, which limits its translational relevance to clinical applications. In this study, we performed small RNA sequencing combined with cell biology studies in primary human trophoblast and 2D human trophoblast stem cell models and revealed that the upregulation of the *SOX4* (SRY-box transcription factor 4) and miR-103a-3p induced by activin A contributes to trophoblast invasion and potential extravillous differentiation. The bioinformatic analysis of proteomic and microRNA profiles from public databases revealed increased expression of the activin A protein and exosomal miR-103a-3p in maternal blood during the second trimester of pregnancy complicated by preeclampsia. Overall, our integrated approach reveals the regulatory mechanism by which activin A, *SOX4*, and miR-103a-3p regulate human trophoblast invasion and EVT differentiation, highlighting their potential as early diagnostic biomarkers for preeclampsia.

## Introduction

The placenta is a temporary and multifaceted organ that connects the fetal and maternal circulation during pregnancy. Placental functions are executed primarily by a specialized group of cells called trophoblasts. In human placentas, progenitor cytotrophoblasts (CTBs) differentiate into either syncytiotrophoblasts (SCTs) or extravillous trophoblasts (EVTs) through two pathways: the villous and extravillous pathways [1]. Compared with other hemochorial placental mammals (i.e., rodents and nonhuman primates), the human placenta exhibits a higher degree of invasiveness [2]. The invasion of human placental trophoblasts is crucial for maintaining a normal pregnancy. EVTs possess a significant invasive ability [3]. For example, EVTs deeply invade the maternal decidua, remodel maternal spiral arteries to maintain adequate fetal nutrition and gas supply, and coordinate the metabolic needs of the placenta [1, 4]. Overall, aberrant trophoblast invasion is associated with impaired placental function, pregnancy complications, and often, early-onset preeclampsia [5, 6].

In recent years, the abnormal expression of microRNAs (miRNAs) in the placentas and blood of patients with preeclampsia has been widely reported. These miRNAs include placenta-specific miRNAs such as the chromosome 19 miRNA cluster (C19MC) [7, 8], maternally expressed miRNAs such as the chromosome 14 miRNA cluster (C14MC) [9], and nonspecific miRNAs (miR195, miR-210, etc.) [10, 11]. These miRNAs act primarily as epigenetic modifiers that are involved in the development of preeclampsia [12, 13]. Specifically, several miRNAs regulate trophoblast function through the modulation of TGF-β superfamily ligands and receptors, including activin type 2A receptors [14].

Activin A is a disulfide-linked homodimer of the inhibin β subunit (βA-βA), a key growth factor of the TGF-β superfamily, and is involved in the regulation of EVT invasion by modulating EMT associated genes [15, 16]. Compared with activin B (βB-βB), activin AB (βA-βB), activin C (βC-βC), and other TGF-β family members, activin A levels have been found to be significantly elevated in women with preeclampsia [7, 17, 18]. The development of preeclampsia is hypothesized to be strongly linked to the abnormal invasion ability of trophoblasts, suggesting that the abnormal expression of activin A and miRNAs may be closely associated with the imbalanced trophoblast invasive ability in preeclampsia. However, systematic studies on the effects of activin A and miRNAs on trophoblast invasion are limited, and the trophoblast cell lines (HTR8/SVneo, JAR, JEG-3, etc.) used in previous studies have shown significant limitations [19].

In this study, we comprehensively analyzed and investigated how activin A regulates trophoblast invasion through miRNAs. Specifically, we used small RNA sequencing (RNA-seq) to profile miRNA expression in activin A-treated first-trimester primary human trophoblasts and identified miR-103a-3p as a core miRNA that promotes trophoblast invasion by activin A. Additionally, we discovered that *SOX4* and miR-103a-3p regulate human trophoblast stem cell (hTSC)-derived EVT invasion by controlling the expression of the miRNA host gene pantothenate kinase 2 (*PANK2*). In summary, this study elucidates the regulatory network of activin A and miRNAs and defines the specific interactive expression pattern and proinvasive role of the transcription factor *SOX4* and miR-103a-3p in both first-trimester primary trophoblasts and hTSC-derived EVTs.

## Materials and methods

### Tissue collection

Placental tissues were obtained with the approval of the Research Ethics Board of the University of British Columbia (certificate #H07-01149) and the Institutional Ethics Committee of the Women’s Hospital, School of Medicine, Zhejiang University (certificate No. 20180113). First-trimester placental villus samples (gestational ages ranging from 6 to 9 weeks) were collected from women who provided written informed consent and who underwent elective surgical termination of pregnancy in the Comprehensive Abortion and Reproductive Education (CARE) program at the BC Women’s Hospital and Health Center in Vancouver, British Columbia, Canada, and the Women’s Hospital, School of Medicine, Zhejiang University, China.

### Primary trophoblast isolation and culture

Primary human trophoblasts were prepared from first-trimester placental villus tissue (6–9 weeks of gestation). Briefly, placental villus tissues were washed with cold PBS (Gibco, Carlsbad, CA, USA), and blood clots were removed. The villi tissues were subsequently dissected, scraped, and mechanically minced into 1–2 mm fragments. Villous fragments were suspended in cold PBS to remove the placental debris. The remaining villus fragments were cultured in DMEM (Gibco) supplemented with 10% fetal bovine serum (Gibco), 100 U/mL penicillin, and 100 μg/mL streptomycin (Gibco) and incubated at 37 °C in a CO_2_ atmosphere with medium changes every other day. After an additional 2 weeks to facilitate trophoblast outgrowth, the trophoblast cells surrounding the villus fragments were collected by trypsinization, as previously described [20]. Primary trophoblasts from passages 2 to 4 were used in this study after validation by immunofluorescence staining for the trophoblast markers HLA-G and KRT7. Each sample was independently isolated from different patients and considered as one biological replicate (*n* = 1). The experiments using the primary trophoblast model were replicated with cells from at least five different patients (*n* = 5).

### Human trophoblast stem cells

#### Stem cell culture

hTSC line CT29 (RCB4937) and CT30 (RCB4938), which were generously provided by Dr. Alexander G Beristain, were established from cytotrophoblasts isolated from the first-trimester placenta by Okae et al., and the cells were cultured as described previously [21]. In general, CT29/CT30 cells were seeded in 5 µg/mL human collagen IV (Sigma-Aldrich, Saint Louis, MO, USA)-precoated 100 mm cell culture dishes and cultured with complete TS cell culture medium containing DMEM/F12 (Gibco), 0.3% bovine serum albumin (BSA, Sigma-Aldrich), 0.5% penicillin/streptomycin (P/S, Gibco), 0.2% (vol/vol) fetal bovine serum (FBS, Gibco), 100 μM 2-mercaptoethanol (2-ME, Gibco), a 1% insulin-transferrin-selenium-ethanolamine solution (ITS-X, Gibco), 1.5 μg/mL L-ascorbic acid (Sigma), 50 ng/mL epidermal growth factor (EGF, Peprotech, Cranbury, NJ, USA), 2 μM CHIR99021 (Sigma-Aldrich), 0.5 μM A83-01 (Sigma-Aldrich), 1 μM SB431542 (Peprotech), 0.8 mM valproic acid (VPA, Sigma-Aldrich), and 5 μM Y27632 (Tocris, Bristol, England, UK).

#### hTSC-derived EVT culture

CT29/CT30 cells were plated onto 600 mm plates precoated with 1 μg/mL human collagen IV at a density of 2.5 × 10^5^ cells per dish. The cells were initially cultured in ice-cold EVT differentiation basic medium containing DMEM/F12, 100 μM 2-mercaptoethanol, 0.5% P/S, 0.3% BSA, 7.5 μM A83-01, and 2.5 μM Y27632 supplemented with 100 ng/mL neuregulin-1 (NRG1; Cell Signaling Technology, Danvers, MA, USA), 4% knockout serum replacement (KSR; Thermo Fisher, Waltham, MA, USA), and 2% growth factor-reduced Matrigel (Corning, Somerville, MA, USA). On Day 3, the culture medium was changed to EVT Day 3 culture medium consisting of EVT differentiation basic medium, 4% KSR, and 0.5% growth factor-reduced Matrigel. On culture Day 6 of EVT cell differentiation, the medium was replaced with basic EVT differentiation medium supplemented with 0.5% Matrigel. The cells were cultured for another 2–4 days, and differentiation was stopped on either Day 8 or Day 10. Each hTSC line (CT29 and CT30) of different passages was differentiated as one (*n* = 1) replicate. For the experiments using the hTSC models, cell differentiation was performed three (*n* = 3) independent times.

#### Transcriptomic analysis using small RNA sequencing

Total RNA was extracted from primary human trophoblasts (*n* = 4 placentas) treated with the vehicle control (4 mM HCl containing at least 0.1% bovine serum albumin) or activin A (50 ng/mL diluted in 4 mM HCl containing at least 0.1% bovine serum albumin; R&D Systems, Minneapolis, MN, USA) for 24 h with TRIzol (Thermo Fisher) reagent according to the manufacturer’s instructions. The library was prepared with 1 μg of total RNA that was purified by electrophoretic separation, and small RNA regions corresponding to the 18-30 nt bands in the marker lane (14-30 ssRNA Ladder Marker, TAKARA, Kusatsu, Japan) were excised, recovered, and subsequently transcribed into cDNA using SuperScript II Reverse Transcriptase (Thermo Fisher). Following several rounds of PCR amplification with the PCR Primer Cocktail and PCR Mix, the PCR products were selected by agarose gel electrophoresis with target fragments of 110–130 bp. Then, the 18-30 nt small RNAs were transcribed into cDNAs, selected by agarose gel electrophoresis, and purified using a QIAquick Gel Extraction Kit (QIAGEN, Valencia, CA). The final ligation PCR products were sequenced using the BGISEQ-500 platform (BGI, Beijing Genomics Institution). The raw tags were mapped to the reference genome (GRCh38.p12) and other sRNA databases, including miRbase, siRNA, piRNA, and snoRNA, after which they were processed into clean tags according to standard protocols [22]. Fragments per kilobase of exon per million reads (FPKMs) were calculated, and the differential expression analysis was performed using DEGseq [23], with an absolute value of a Log2 (fold change) cutoff of ≥1 and a *Q* value cutoff of ≤0.001 as the thresholds for significance. The small RNA-seq data were plotted in a heatmap using the ggplot2 and ComplexHeatmap R packages. The Gene Ontology (GO) enrichment analysis, KEGG pathway classification, pathway enrichment analysis of target genes, and enrichment of miRNA-target interactions were conducted using the R function hyper and the Path DIP tool [24]. The top 20 differentially expressed miRNAs (based on the *Q* value) were analyzed using the miRNet2.0 platform (miRTarBase v9.0) with the instructions filtered by the shortest path [25].

### RNA isolation and reverse transcription quantitative real-time PCR

#### Total RNA

Total RNA was isolated using TRIzol according to the manufacturer’s protocol. Two micrograms of total RNA were transcribed using dNTPs, random primers, 5× RT buffer, and Moloney murine leukemia virus (M-MLV) reverse transcriptase (Promega, Madison, WI, USA). The synthesized cDNA was subjected to a quantitative real-time PCR analysis using 1×SYBR Green Master Mix (Applied Biosystems, Foster City, CA, USA) and an Applied Biosystems QuantStudio3 system. The specificity of each assay was confirmed by analyzing a dissociation curve, and each sample was assayed in duplicate. The relative quantification of mRNA levels was performed using the comparative quantification cycle (Cq) method, with glyceraldehyde-3-phosphate dehydrogenase (GAPDH) serving as the reference gene and using the formula 2^−ΔΔCq^. The primers used are summarized in Table S1.

#### Small RNA

Small RNA was isolated using the mirVana miRNA isolation kit without phenol (Thermo Fisher) according to the manufacturer’s instructions, and mature miRNA was converted to cDNA using the TaqMan miRNA reverse transcription kit (Thermo Fisher) and miRNA-specific stem-loop reverse transcription primer. This step was followed by a real-time PCR analysis with paired specific TaqMan miRNA Assay probes using TaqMan Universal Master Mix II no UNG (Thermo Fisher, Cat# 4440043) and an Applied Biosystems QuantStudio3 system. The miRNA-specific primers and TaqMan probes used are summarized in Table S2.

#### Small interfering RNA (siRNA) transfection

Cells were transfected with 25 nM ONTARGETplus SMARTpool siRNA (Dharmacon, Lafayette, CO, USA) targeting the genes in primary trophoblasts and 50 nM in hTSC-derived EVTs using Lipofectamine™ RNAiMAX (Thermo Fisher, Cat# 13778075) to silence endogenous *SMAD2*, *SMAD3*, *SMAD4*, and *SOX4* in primary trophoblasts, as well as *SOX4* in hTSC-derived EVTs. The siCONTROL nontargeting pool (Dharmacon) was used as the transfection control.

#### miRNA Mimic/Inhibitor Transfection

An miR-103a-3p mimic (Thermo Fisher, Cat# 4464066, Assay ID-MC10632) was transfected into trophoblasts using Lipofectamine RNAiMAX at a concentration of 5 nM to achieve the forced expression of miR-103a-3p. As a control, cells were transfected with the miRNA positive control (Thermo Fisher) at the same concentration. Cells were transfected with 50 nM 103a-3p inhibitor (Thermo Fisher, Cat# 4464084, Assay ID-MH10632) using Lipofectamine RNAiMAX to knock down the expression of miR-103a-3p, and the miRNA negative control was used as the negative transfection control (Thermo Fisher).

#### Immunofluorescence staining

The cells were cultured on chamber slides or Transwell inserts, fixed, and permeabilized with methanol for 20 min at −20 °C. The cells were blocked with serum-free protein blocking solution (X0909, Dako, Carpinteria, CA, USA) for 1 h at room temperature and then incubated with the indicated primary antibodies overnight at 4 °C: mouse monoclonal anti-human leukocyte antigen-G (HLA-G, 1:100, clone 4H84, Cat#11-499-C100; ExBio, Amsterdam, the Netherlands), mouse monoclonal anti-cytokeratin 7 (KRT7, 1:100, RCK105, Cat# sc-23876; Santa Cruz Biotechnology, Dallas, Texas, USA), or mouse monoclonal anti-SOX4 (1:100; Santa Cruz Biotechnology, Cat# sc-518016). Following an overnight incubation, the cells on the slides were washed with PBST 3 times, incubated for 1 h at room temperature with an Alexa Fluor® goat anti-mouse 594-conjugated secondary antibody (1:500, Cat# A-11005; Life Technologies, Thermo Fisher) diluted in 1× PBST supplemented with 0.1% BSA and mounted with ProLong Gold mounting media containing DAPI (Life Technologies).

#### Western blot

The cells were lysed in a cold solution consisting of lysis buffer (Cell Signaling Technology), phenylmethanesulfonylfluoride (PMSF), and a protease inhibitor cocktail (Sigma-Aldrich). The normalization of unknown protein concentrations used for downstream sodium dodecyl sulfate-polyacrylamide gel electrophoresis (SDS-PAGE) was determined using a DC protein assay (Bio-Rad Laboratories, Hercules, CA, USA), and the absorbance was recorded at 650 nm with a SpectraMax iD3 plate reader (Molecular Devices, San Jose, CA, USA). Equal amounts of protein were separated via SDS-PAGE and transferred onto polyvinylidene fluoride (PVDF) membranes (Bio-Rad Laboratories). The PVDF membranes were blocked with 5% w/v nonfat dry milk diluted in 1× Tris-buffered saline (TBS) supplemented with 0.1% (vol/vol) Tween-20 (TBST) for 1 h at room temperature and then incubated with the following primary antibodies diluted in 1× TBST containing 5% w/v nonfat dry milk overnight at 4 °C with gentle shaking: rabbit anti-phospho-SMAD2 (1:1000; Cell Signaling, Cat# 3180), rabbit anti-phospho-SMAD3 (1:1000; Cell Signaling, Cat# 9520), mouse anti-SMAD2 (1:1000; Cell Signaling, Cat# 3103), rabbit anti-SMAD3 (1:1000; Cell Signaling, Cat# 9523S), rabbit anti-SMAD4 (1:1000; Cell Signaling, Cat# 38454), mouse anti-SOX4 (1:600; Santa Cruz Biotechnology, Cat# sc-518016), and mouse anti-GAPDH (1:1000; Santa Cruz Biotechnology, Cat# sc-365062). After the overnight incubation with the primary antibody, the membranes were washed three times for 15 min with 1× TBST and incubated with anti-mouse or anti-rabbit horseradish peroxidase (HRP)-conjugated goat secondary antibodies (1:1000; Bio-Rad, anti-mouse: Cat#170-6516, anti-rabbit: Cat#170-6515) for 1 h at room temperature. The membrane-bound HRP was washed three times for 15 min with 1× TBST. The membrane was incubated with a mixture of Pierce ECL Western blotting Substrate (Thermo Fisher, Cat# 32016) and SuperSignal West Femto Maximum Sensitivity Substrate (Thermo Fisher, Cat# 34096) and exposed to X-ray film. The intensities of the bands were quantified with ImageJ (National Institutes of Health) software. Images have been cropped for presentation purposes. Full-size Western blot images are available in the Supplemental Material.

### Matrigel transwell cell invasion assay

#### Primary trophoblast

The invasion of primary trophoblasts was assessed using the Matrigel Transwell invasion assay, as described in our previous study [26]. The MTT assay was used to measure the cell variability before invasion assay (Recorded absorbance at 490 nm). First, the Matrigel chambers were prepared with 40 μL of a diluted growth factor-reduced Matrigel basement membrane matrix solution (1 mg/mL diluted in DMEM supplemented with 2% FBS; Corning) on top of Transwell tissue culture plate polyester inserts (6.5 mm with 8-μm pore size, 24 wells, VWR, Radnor, PA, USA), and then, 1 × 10^5^ cells resuspended in 250 μL of DMEM supplemented with 0.1% FBS were added to the upper chamber of the Transwell inserts, and 750 μL of medium with 10% FBS was added to the lower chamber. The inserts were collected after the primary trophoblasts were cultured in the insert chamber for 24 h. Noninvading cells were removed from the upper side of the membrane with a cotton swab. The cells that penetrated the membrane were fixed with cold methanol at −20 °C for 20 min, stained with crystal violet (0.5%, Sigma-Aldrich) for 30 min, and subsequently washed thoroughly with PBS. The Transwell insert membrane was subsequently cut with a blade and mounted on a slide with the underside of the membrane facing upward under a glass coverslip. Images of the Transwell assay were captured with an Olympus BX61 Automated Fluorescence and Transmittance Upright microscope (Olympus, Japan) and cellSens Dimension software (Olympus) with a 4x objective. The analyzed particle function of the ImageJ software was used to count the number of invaded cells.

#### Trophoblast stem cells

Transwell inserts were coated with growth factor-reduced Matrigel basement membrane matrix (3.33 mg/mL diluted in serum-free DMEM/F12). Then, 1 × 10^5^ CT29 cells were seeded in the upper chamber of the Transwell inserts with EVT differentiation medium. The differentiation medium was then changed as described above on Days 3 and 6 in both the upper and lower insert chambers, with the addition of 10% FBS to the lower chamber beginning on Day 6. The inserts were collected on Day 8 and subsequently stained with either crystal violet or antibodies for immunofluorescence staining, as described previously.

#### Cell clustering, identification, and trophoblast sub-setting

The codes used for the integrated analysis of the datasets GSE174481 and E-MTAB-6701 for cell clustering, identification, and trophoblast sub-setting are available at https://github.com/MatthewJShannon. Based on the integrated analysis, the code for analyzing *SOX4* and *HLA-G* gene expression in first-trimester trophoblast states and distinguishing differences in *SOX4* expression by placental sex is available at https://github.com/JIAMIN-XIE.

### Statistical analysis

The data are presented as the means with standard deviations. All calculations were performed using GraphPad Prism9 (GraphPad Software). Two-tailed unpaired Student’s *t* test was used for comparisons between two groups. Multiple group comparisons were analyzed by one-way analysis of variance (ANOVA) followed by Tukey’s multiple comparisons test. The differences were considered significant at *p* < 0.05. Investigators were not blinded to allocation during the experiments. The sample size of human tissue-derived cells was determined based on prior experience, and no randomization was used.

## Results

### Expression profile of miRNAs induced by activin A in primary human trophoblasts

We isolated primary human trophoblasts from the chorionic villi of first-trimester placentas (between 6–9 weeks of gestation) as previously described [20] and treated the cells with exogenous activin A for 24 h before performing small RNA-seq. The primary trophoblast identity was assessed using immunofluorescence staining with antibodies against known trophoblast markers (Fig. S1). A total of 1657 miRNAs were detected within the small RNA-seq data. These miRNAs included 98 differentially expressed miRNAs (the expression of 85 miRNAs decreased, and the expression of 13 miRNAs increased; *Q* value ≤0.001, absolute value of Log2 (fold change) ≥1) identified by DEGseq [23] (Fig. S2A). The Gene Ontology (GO) analysis revealed that the target genes of the differentially expressed miRNAs are involved in various biological processes, including protein binding, nucleotide binding, and transferase activity, with their products predominantly located in the cytoplasm (Fig. S2B). Moreover, the GO analysis indicated the enrichment of molecular functions related to multicellular organism development, phosphorylation, cell cycle regulation, and cell differentiation. The KEGG pathway classification analysis demonstrated that miRNA-targeted genes are enriched in processes such as cell motility, signal transduction, and the development of cancers (Fig. S2C).

**Fig. 1:**
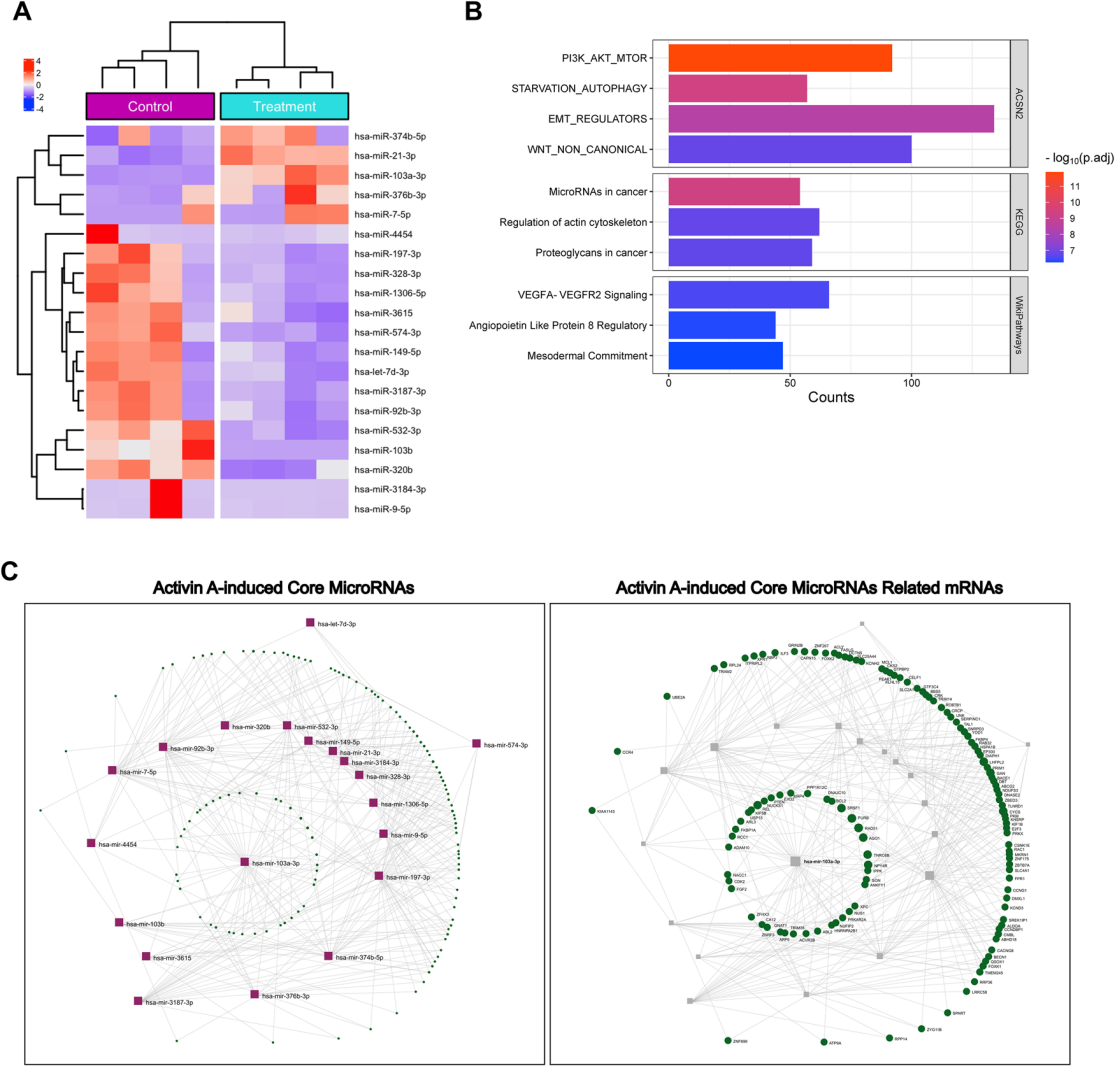
miR-103a-3p functions as a focal node in the activin A-induced miRNA-mRNA network. **A** Heatmap of the hierarchical clustering analysis of small RNA-seq data displaying the top 20 differentially expressed miRNAs (ranked by the *Q* value) between vehicle control-treated primary trophoblasts and activin A (50 ng/mL)-treated primary trophoblasts (rows show the *Z* scores). **B** Top 10 enriched pathways associated with the top 20 differentially expressed miRNAs induced by activin A treatment in primary trophoblasts. The pathway enrichment analysis was performed with target genes (mRNAs) of the top 20 miRNAs. **C** The miRNA-mRNA interaction network was constructed by analyzing the top 20 differentially expressed miRNAs with miRNet2.0. The left panel shows the interactions among miRNAs. The right panel highlights the mRNAs targeted by the top 20 differentially expressed miRNAs.

We then conducted a pathway enrichment analysis including the top 20 identified activin A-induced miRNAs (based on the *Q* value) using pathDIP [24] (Fig. 1A). Notably, EMT regulators and Wnt signaling, PI3K signaling, and VEGF signaling components (all of which are known to be important in the differentiation and invasion of human trophoblasts) were highly enriched among the top 20 differentially expressed activin A-induced miRNAs (Fig. 1B). The miRNet2.0 platform [25] was used to construct an interaction network based on the degree and betweenness of these miRNAs and their targets to further investigate the interactions of the top 20 differentially expressed miRNAs and potential target genes (Table S3). miR-103a-3p was predicted to be the focal miRNA node with the highest degree and betweenness value of the entire network. These findings suggest an important and central regulatory role of miR-103a-3p in the activin A-induced miRNA-mRNA network (Fig. 1C).

### miR-103a-3p contributes to activin A-induced primary human trophoblast invasion

**Fig. 2:**
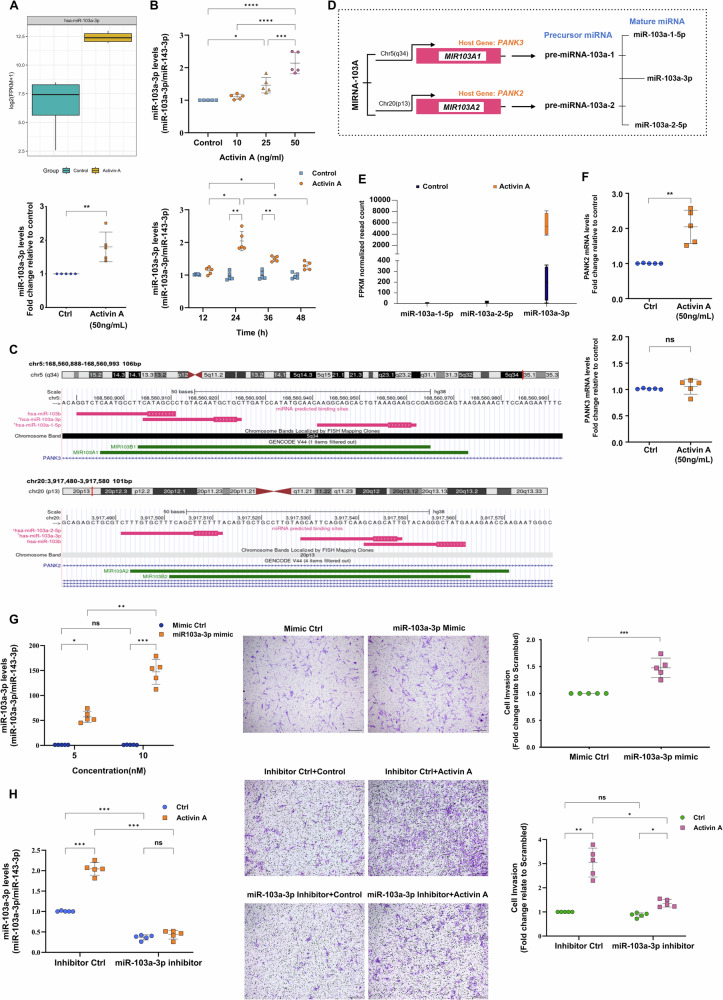
miR-103a-3p contributes to activin A-induced primary trophoblast invasion. **A** The upper panel shows a box plot of miR-103a-3p expression in vehicle control and activin A-treated first-trimester placental trophoblasts obtained from small RNA-seq data (*n* = 4 placentas). The lower panel shows the RT-qPCR validation of the activin A-induced increase in miR-103a-3p expression in primary trophoblasts treated with or without 50 ng/mL activin A for 24 h. The data were normalized to those of the endogenous control miR-143-3p. Statistical analyses of differences between two groups were performed using Student’s *t* test; significance was defined as *p* < 0.05. Each condition was performed in five independent experiments (*n* = 5). **B** The upper panel shows the expression of miR-103a-3p in first-trimester placental trophoblasts following treatment with the vehicle control or activin A at different concentrations (10, 25, or 50 ng/mL) for 24 h. The lower panel shows the expression levels of miR-103a-3p in first-trimester placental trophoblasts following treatment with either the vehicle control or 50 ng/mL activin A for the indicated durations, with the expression normalized to that in the 12-h control condition. The miR-103a-3p expression levels were analyzed by RT-qPCR. Statistical analyses of the differences between groups were performed using ANOVA and Tukey’s multiple comparisons test; significance *p* < 0.05. Each condition was performed in five independent experiments (*n* = 5). **C** Chromosomal locations and sequences of miR-103a-3p. *MIR-103A1* and *MIR103A2* are mapped to chr5 (q34) and chr20 (p13), and the genes encoding *MIR103A1*/*MIR103A2* are *PANK3* and *PANK2*, respectively. **D** Schematic representation of the production of mature miR-103a-3p from the host genes. **E** Comparison of the expression of miR-103a-3p, miR-103a-1-5p, and miR-103a-2-5p in vehicle control- and activin A-treated first-trimester placental trophoblasts based on the small RNA-seq data (*n* = 4 placentas). **F** RT-qPCR validation of *PANK2* and *PANK3* expression in primary trophoblasts treated with or without 50 ng/mL activin A for 24 h. The data were normalized to those of GAPDH. Statistical analyses of differences between two groups were performed using Student’s *t* test; significance was defined as *p* < 0.05. Each condition was performed in five independent experiments (*n* = 5). **G** First-trimester primary trophoblasts were transfected for 48 h with the unspecific scrambled mimic control or 5 nM or 10 nM miR-103a-3p mimic, and the levels of miR-103a-3p were examined by RT-qPCR (left panel). Statistical analyses of differences between groups were performed using ANOVA and Tukey’s multiple comparisons test; significance *p* < 0.05. Each condition was performed in five independent experiments (*n* = 5). Representative images from Matrigel-coated Transwell invasion assays and scatter plots showing the effect of miR-103a-3p overexpression (5 nM miR-103a-3p mimic) on the invasion of primary trophoblast cells (middle and right panels). Statistical analyses of differences between groups were performed using Student’s *t* test; significance was defined as *p* < 0.05. Each condition was performed in five independent experiments (*n* = 5). **H** Primary trophoblasts were transfected for 48 h with the unspecific scrambled inhibitor control or miR-103a-3p inhibitor (50 nM) prior to treatment with the vehicle control or 50 ng/mL activin A for 24 h. The efficiency of miR-103a-3p knockdown was evaluated via RT-qPCR (left panel). Representative images and summarized quantitative results from Matrigel-coated Transwell invasion assays and scatter plots examining the effect of miR-103a-3p knockdown on activin A-induced invasion are shown in the middle and right panels. Statistical analyses of differences between groups were performed using ANOVA and Tukey’s multiple comparisons test; significance *p* < 0.05. Each condition was performed in five independent experiments (*n* = 5). *p < 0.05; **p < 0.01; and ***p < 0.001. ns not significant. The data are presented as the means ± SDs. Scale bars: 100 μm (**G** and **H**).

The small RNA-seq data revealed that miR-103a-3p was significantly upregulated (Log2 fold change = 5.09, *Q* value < 0.001) upon activin A treatment. The levels of miR-103a-3p in primary trophoblasts with or without activin A treatment were measured to validate the small RNA-seq results. Activin A significantly increased the expression of miR-103a-3p in a time- and dose-dependent manner, according to the RT-qPCR analysis (Fig. 2A, B). The UCSC genome browser [27], featuring the MBS annotation track [28], was subsequently utilized to analyze the chromosomal location, sequence, and binding site of miR-103a-3p (Fig. 2C). miR-103a-3p originates from the primary transcripts (pri-miRNAs) of *MIR103A1* and *MIR103A2*, which are mapped to chr5 (q34) and chr20 (p13), respectively. The host genes are *PANK3*, encoding *MIR103A1*, and *PANK2*, encoding *MIR103A2*. Therefore, following the synthesis of precursor miRNAs premiR-103a-1 and premiR-103a-2 transcribed from either *PANK3* (*MIR103A1*) or *PANK2* (*MIR103A2*), these precursor miRNAs are cleaved into a double-stranded miRNA that differs only in its 5’ strand. This double-stranded miRNA subsequently separates to form a 3’ mature miRNA (miR-103a-3p) or a 5’ mature miRNA (miR-103a-1-5p or miR-103a-2-5p), each of which is 23 nucleotides long (Fig. 2D).

For all samples, the expression levels of miR-103a-1-5p (fragments per kilobase per million [FPKM] < 9) and miR-103a-2-5p (FPKM < 22) were consistently low (Fig. 2E). A comparison of the levels of miR-103a-3p, miR-103a-1-5p, and miR-103a-2-5p suggested that premiR-103a-1 and premiR-103a-2 predominantly give rise to mature miR-103a-3p rather than miR-103a-1/2-5p with or without activin A treatment in the primary trophoblast model. Furthermore, activin A treatment upregulated the expression of the host gene *PANK2* but not *PANK3* in primary trophoblasts (Fig. 2F).

We next investigated the role of miR-103a-3p in primary human trophoblast invasion and its potential involvement in mediating activin A-induced trophoblast invasion using in vitro growth factor-reduced Matrigel Transwell assays. Compared with the control, the transfection of 5 nM miR-103a-3p mimic into primary trophoblasts resulted in a significant increase in miR-103a-3p expression, approximately 40- to 70-fold, and enhanced primary trophoblast invasion (Fig. 2G). Finally, the role of miR-103a-3p in activin A-induced trophoblast invasion was explored via the combination of activin A treatment and a miR-103a-3p inhibitor in primary trophoblasts. Following the transfection of the miR-103a-3p inhibitor into primary trophoblasts, the invasion ability of the cells was not significantly affected. Nevertheless, although not completely effective, the reduction in miR-103a-3p expression significantly inhibited activin A-induced primary trophoblast invasion (Fig. 2H). To investigate potential downstream targets involved in mediating this phenotype, we transfected primary trophoblasts with miR-103a-3p mimics and performed RT-qPCR to measure target gene expression. The results showed that expression of co-targeted genes such as *ANKFY1*, *CA12*, *NUFIP2*, and *PRKAR2A* decreased with miR-103a-3p overexpression (Fig. S3A). Using mirDIP, we identified top target genes and enriched pathways, with *AXIN2*, a key component of Wnt signaling, being prominently highlighted. Validation confirmed that miR-103a-3p significantly inhibits *AXIN2* expression (Fig. S3B, C). Additionally, genes related to EMT, ECM, and cell adhesion, including *EIF1AX*, *CALU*, and *TGFBR3*, were also downregulated following miR-103a-3p transfection (Fig. S3D).

### The regulation of miR-103a-3p by activin A induces canonical SMAD2/3-SMAD4 signaling in primary human trophoblasts

**Fig. 3:**
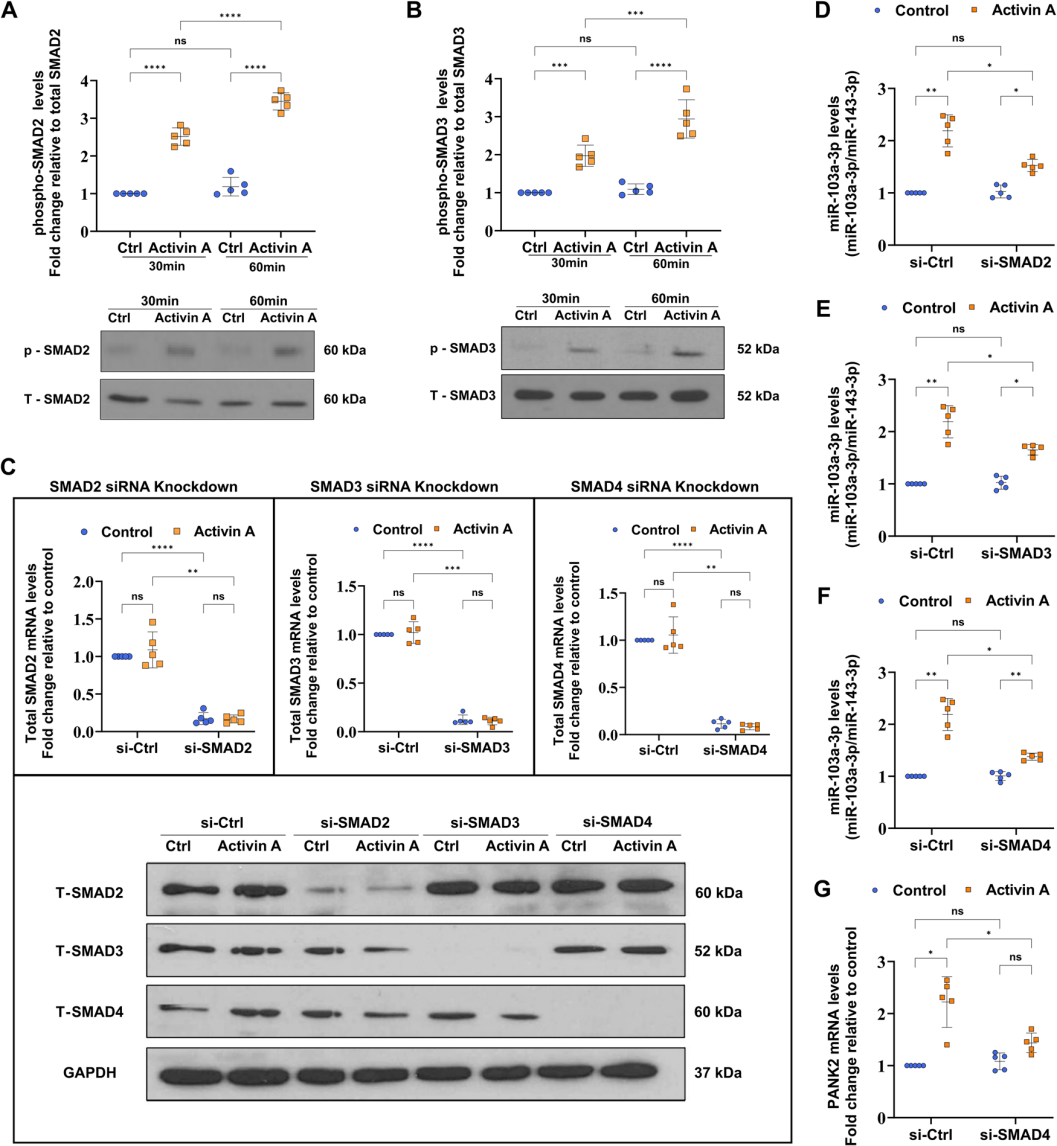
Canonical SMAD2/3-SMAD4 signaling is required for activin A-induced expression of miR103a-3p in primary trophoblasts. **A**, **B** Primary trophoblast cells were treated with vehicle control or 50 ng/mL activin A for 30 or 60 min. The levels of phosphorylated SMAD2 and SMAD3 were examined by Western blotting with specific antibodies. Each membrane was stripped and subsequently probed with an antibody specific to the total SMAD2 or SMAD3. The summarized quantitative results were normalized to the total SMAD2 or SMAD3 levels. Statistical analyses of differences between groups were performed using ANOVA and Tukey’s multiple comparisons test; significance *p* < 0.05. Each condition was performed in five independent experiments (*n* = 5). **C** Primary trophoblasts were transfected with 25 nM scrambled siRNA (si-Ctrl) or SMAD2/3/4 siRNA (si-SMAD2, si-SMAD3, or si-SMAD4) for 48 h prior to treatment with vehicle control or 50 ng/mL activin A for 24 h. The knockdown efficiency was examined by RT-qPCR and Western blotting. The summarized quantified results were normalized to the level of GAPDH. Statistical analyses of differences between groups were performed using ANOVA and Tukey’s multiple comparisons test; significance *p* < 0.05. Each condition was performed in five independent experiments (*n* = 5). **D**, **E** The expression of miR-103a-3p in primary trophoblasts transfected with 25 nM scrambled siRNA Ctrl, SMAD2 siRNA or SMAD3 siRNA was examined by RT-qPCR. The summarized quantified results were normalized to the level of GAPDH. Statistical analyses of differences between groups were performed using ANOVA and Tukey’s multiple comparisons test; significance *p* < 0.05. Each condition was performed in five independent experiments (*n* = 5). **F**, **G** The expression of miR-103a-3p and the host gene *PANK2* in primary trophoblasts silenced with the Ctrl or SMAD4 siRNA was examined by RT-qPCR. The summarized quantified results were normalized to the levels of GAPDH (mRNA) or miR-143-3p (miRNA). Statistical analyses of differences between groups were performed using ANOVA and Tukey’s multiple comparisons test; significance *p* < 0.05. Each condition was performed in five independent experiments (*n* = 5). *p < 0.05; **p < 0.01; and ***p < 0.001. ns not significant. The data are presented as the means ± SDs.

As a method to assess the involvement of the canonical SMAD2/3-SMAD4 signaling pathway in the regulation of miR-103a-3p by activin A, Western blotting was performed to examine the phosphorylation of SMAD2 and SMAD3 in primary trophoblast cells following activin A stimulation for 30 and 60 min (Fig. 3A, B). The increased levels of phosphorylated SMAD2 and SMAD3 suggest that activin A may exert its biological function in primary trophoblasts by activating the downstream SMAD2/3-SMAD4 signaling pathway. Next, we conducted a series of gene silencing experiments using siRNAs targeting *SMAD2*, *SMAD3*, and *SMAD4*. Cells transfected with a nonsilencing siRNA (si-Ctrl) served as the control. Knockdown of the respective siRNAs resulted in a consistent reduction in *SMAD2*, *SMAD3*, or *SMAD4* expression (Fig. 3C). Following the knockdown of the SMAD2/3-SMAD4 signaling pathway, we stimulated primary trophoblast cells with activin A and silenced *SMAD2* or *SMAD3* expression. This experiment resulted in decreased miR-103a-3p expression following treatment with activin A (Fig. 3D, E). In contrast, silencing *SMAD4* resulted in the efficient suppression of the expression of miR-103a-3p as well as the host gene *PANK2* (Fig. 3F, G). While activin A is known to induce miR-103a-3p expression through SMAD2/3-SMAD4-induced *PANK2* expression, the results we observed after the inhibition of miR-103a-3p suggest the involvement of a noncanonical signaling pathway or transcription factor.

### The transcription factor *SOX4* is required for activin A-induced miR-103a-3p expression in primary human trophoblasts

We utilized data from the following publicly available datasets to explore noncanonical signaling pathways or transcription factors involved in activin A-induced miR-103a-3p expression: GSE125642 [29], GSE158295 [30], and GSE111717 [31]. The analysis of GSE125642 suggested that the transcription factor *SOX4* may induce *PANK2* expression in human mammary epithelial cell lines but has no regulatory effect on *PANK3* expression (Fig. S4A). Furthermore, in the GSE158295 dataset, silencing *SOX4* expression in human breast cancer cells resulted in decreased *PANK2* expression, whereas *PANK3* expression remained unchanged (Fig. S4B). Interestingly, the GSE111717 dataset showed that a relatively low concentration of activin A (10 ng/mL) promoted *SOX4* expression in human embryonic stem cells (Fig. S4C). Overall, these findings of transcriptional regulation by *SOX4* echo the activation of the SMAD2/3-SMAD4 signaling pathway by activin A to induce the expression of miR-103a-3p from the host gene *PANK2* but not *PANK3* in primary human trophoblasts.

**Fig. 4:**
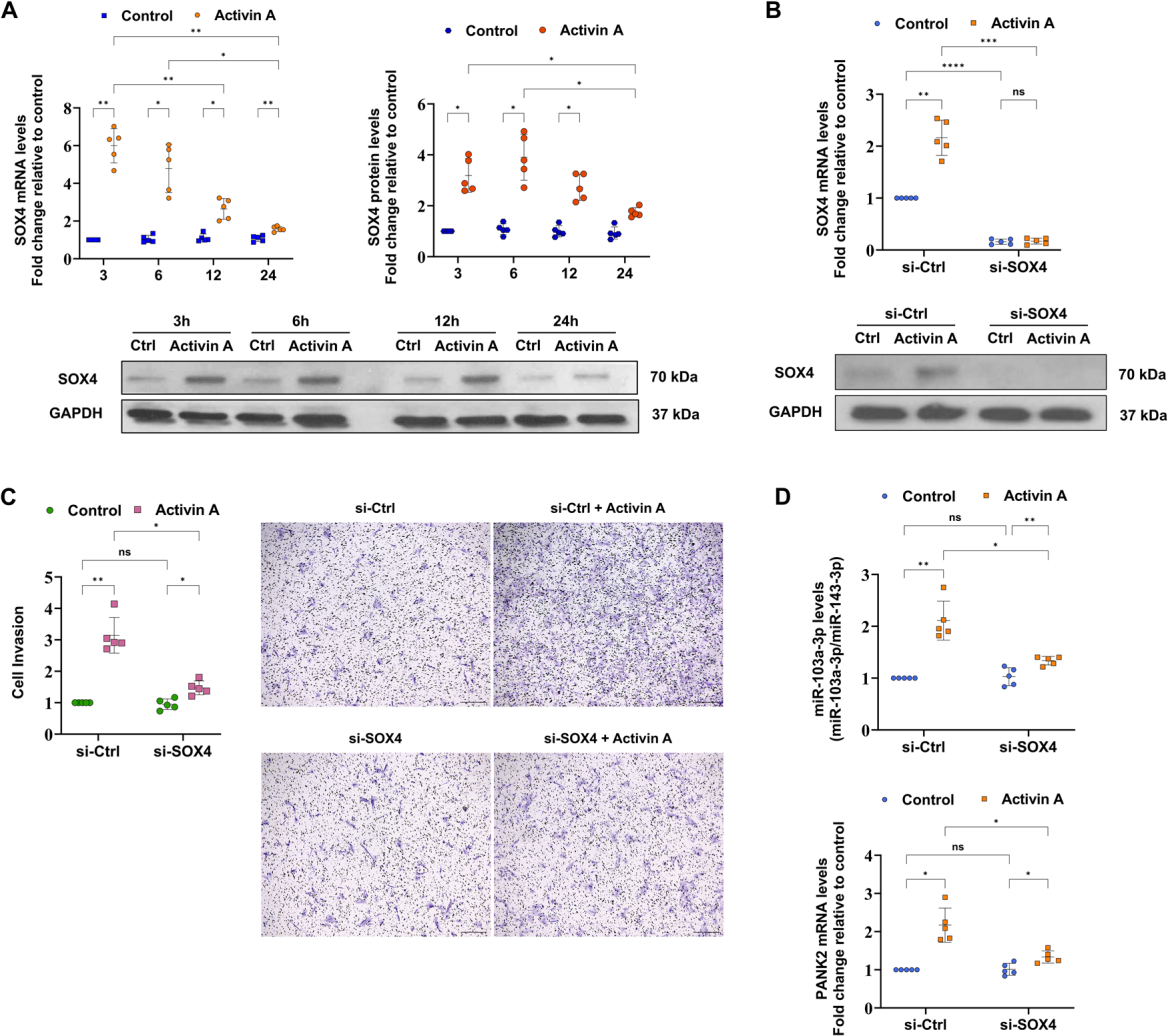
The transcription factor *SOX4* contributes to activin A-induced invasion and increases miR-103a-3p expression. **A** First-trimester primary trophoblasts were treated with the vehicle control or 50 ng/mL activin A for 3, 6, 12, or 24 h, as indicated. *SOX4* mRNA expression levels were analyzed by RT-qPCR, normalized to the level of GAPDH, and reported with respect to the 3 h control. Representative images of SOX4 protein levels analyzed by Western blotting, with GAPDH used as the loading control. Statistical analyses of differences between groups were performed using ANOVA and Tukey’s multiple comparisons test; significance *p* < 0.05. Each condition was performed in five independent experiments (*n* = 5). **B**, **C** Primary trophoblasts were transfected with 25 nM scrambled siRNA (si-Ctrl) or SOX4 siRNA (si-SOX4) for 48 h prior to treatment with the vehicle control or 50 ng/mL activin A for 24 h. The knockdown efficiency was examined by RT-qPCR and Western blotting. The summarized quantified results were normalized to the level of GAPDH. Representative images and summarized quantitative results from Matrigel-coated Transwell invasion assays examining the effect of *SOX4* knockdown on activin A-induced invasion are shown. Statistical analyses of differences between groups were performed using ANOVA and Tukey’s multiple comparisons test; significance *p* < 0.05. Each condition was performed in five independent experiments (*n* = 5). **D** The expression of miR-103a-3p and the host gene *PANK2* in primary trophoblasts silenced with a scrambled or SOX4 siRNA was examined by RT-qPCR. The summarized quantified results were normalized to the levels of GAPDH (mRNA) or miR-143-3p (miRNA). Statistical analyses of differences between groups were performed using ANOVA and Tukey’s multiple comparisons test; significance *p* < 0.05. Each condition was performed in five independent experiments (*n* = 5). *p < 0.05; **p < 0.01; and ***p < 0.001. ns not significant. The data are presented as the means ± SDs.

Based on the findings described above, the expression of the *SOX4* mRNA and the SOX4 protein was determined following exogenous stimulation with activin A. The data revealed that *SOX4* expression increased from 3 h after stimulation and continued to increase until 24 h, indicating a time-dependent regulatory effect of activin A on *SOX4* expression (Fig. 4A). We first silenced *SOX4* expression using specific on-target siRNAs and then stimulated primary human trophoblasts with activin A before conducting in vitro growth factor-reduced Matrigel Transwell assays to examine how the transcription factor SOX4 mediates activin A-promoted trophoblast invasion and its regulatory effects on activin A-induced miR-103a-3p expression. The results showed that *SOX4* knockdown significantly attenuated activin A-induced trophoblast invasion (Fig. 4B, C), indicating that *SOX4* is essential for the proinvasive effects of activin A. Moreover, the silencing of *SOX4* expression concurrently affected the activin A-induced expression of miR-103a-3p and the host gene *PANK2* (Fig. 4D). Together, these findings reveal the regulatory mechanism of *SOX4* in activin A-induced trophoblast invasion and miR-103a-3p expression. Importantly, we show for the first time that *SOX4* functions as an activin A-induced transcription factor in human trophoblast invasion.

### *SOX4* contributes to the acquisition of invasiveness in hTSC-derived EVTs by mediating miR-103a-3p expression

**Fig. 5:**
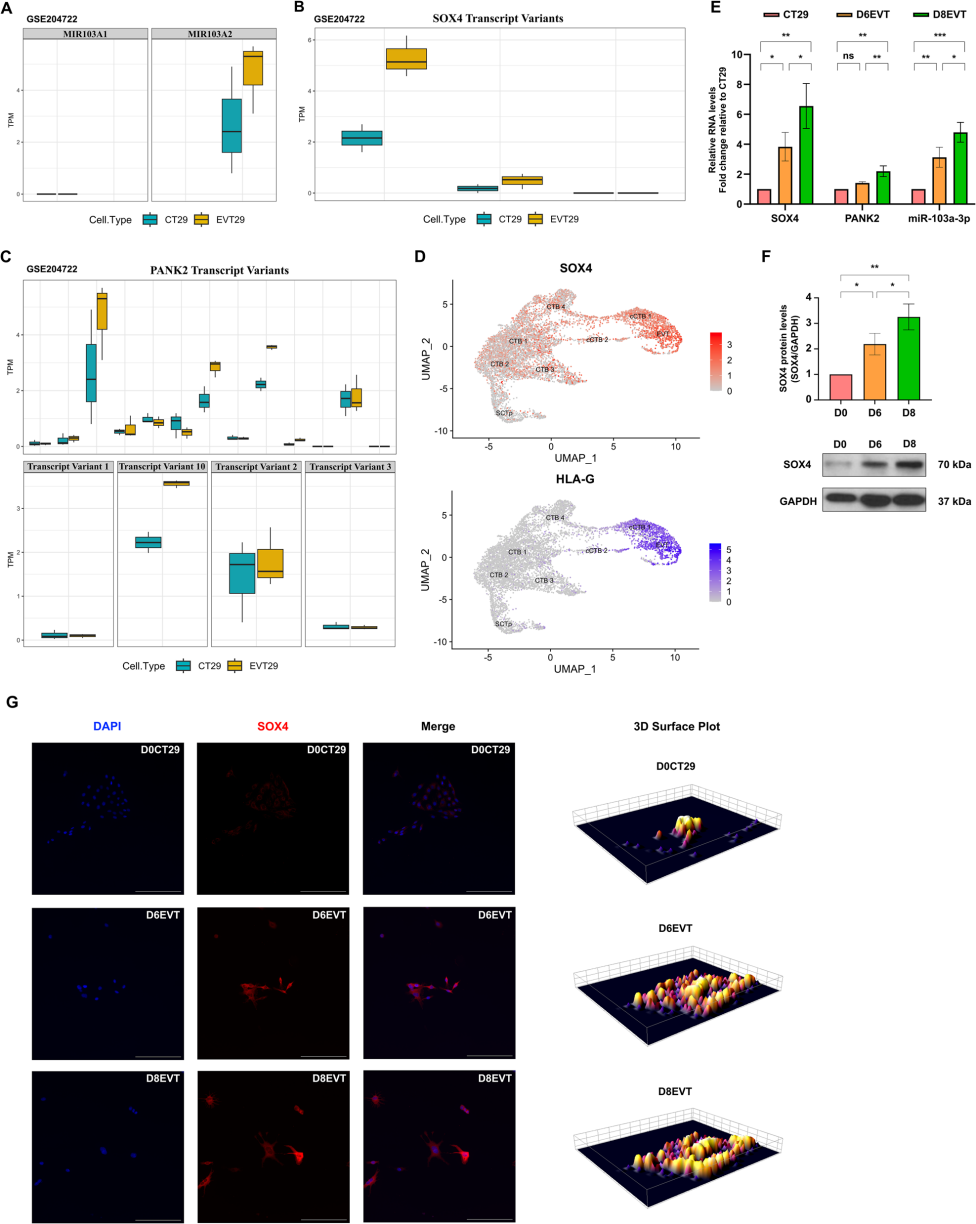
The expression patterns of *SOX4*, *PANK2*, and miR-103a-3p in the different states of CT29 hTSCs. **A** Box plot showing the expression levels of the *MIR103A1* and *MIR103A2* transcripts in the CT29 hTSC stem cell state (CT29) and EVT state (EVT29). **B** Box plot showing the expression levels of *SOX4* transcript variants in the CT29 stem cell state and EVT29 state. **C** Box plots showing the expression levels of all *PANK2* transcript variants in the CT29 stem cell state and EVT29 state (upper panel) and the expression levels of four *PANK2* transcript variants relevant to *MIR103A2* transcription identified in Fig. 2C from the UCSC genome browser (lower panel). The data were generated from the RNA-seq dataset GSE204722 (**A**–**C**). The expression levels are presented as TPM-normalized expression values, means ± SDs (*n* = 3). EVT29 represents EVT cells derived from CT29 cells collected on Day 8 of differentiation. **D** UMAP plots displaying *SOX4* (red, upper panel) and HLA-G (blue, lower panel) expression levels in integrated first-trimester trophoblast clusters from placentas of different sexes. The data were generated from the integrated single-cell RNA-seq dataset GSE174481 (placentas, *n* = 7) and the public repository ArrayExpress E-MTAB-6701 (placentas, *n* = 4; decidua, *n* = 4). The cell clusters were labeled according to the authors’ pipeline. **E** Representative images of mRNA or miRNA expression levels of *SOX4*, *PANK2*, and miR-103a-3p in CT29 hTSCs on Days 0, 6, and 8 of differentiation toward EVTs analyzed by RT-qPCR and normalized to those of GAPDH (mRNA) or RNU6B (miRNA). Statistical analyses of differences between groups were performed using ANOVA and Tukey’s multiple comparisons test; *p* < 0.05. Each condition was performed in three independent experiments (*n* = 3). **F** Representative images showing the SOX4 protein levels in CT29 cells on Days 0, 6, and 8 of differentiation toward EVT, as examined by Western blotting with GAPDH as the loading control. Statistical analyses of differences between groups were performed using ANOVA and Tukey’s multiple comparisons test; *p* < 0.05. Each condition was performed in three independent experiments (*n* = 3). **G** Images of immunofluorescence staining of CT29 hTSCs on Days 0, 6, and 8 of differentiation toward EVTs with a specific SOX4 antibody, and the nuclei were stained with DAPI (left panel). The reconstructed 3D surface plot images represent the visualization of the fluorescence intensity of pixels for the SOX4 signal on Day 0, Day 6, and Day 8 converted from the corresponding image of SOX4 immunofluorescence in the left panel (right panel). *p < 0.05; **p < 0.01; and ***p < 0.001. ns not significant. The data are presented as the means ± SDs. Scale bars: 200 μm (**G**).

The RNA-seq analysis of the GSE204722 [32] dataset revealed that *MIR103A1* and *MIR103A2* expression differed during EVT differentiation between the CT27 and CT29 hTSC cell lines. Our analysis of the GSE204722 dataset revealed that the *MIR103A1* transcript was not expressed in either the stem cell state or Day 8 EVT-differentiated cells in either the CT27 or the CT29 cell lines. *MIR103A2* expression was lower in EVT-differentiated CT27 cells but higher in EVT-differentiated CT29 cells than in their respective undifferentiated counterparts (Figs. 5A and S5A). Furthermore, *SOX4* expression patterns mirrored those of *MIR103A2* during EVT differentiation in both the CT27 and CT29 cell lines (Figs. 5B and S5B). The *MIR103A2* transcript is transcribed from the host gene *PANK2*, and the results showed that the overall expression of *PANK2* decreased in EVT-differentiated CT27 cells but increased in EVT-differentiated CT29 cells (Figs. 5C and S5C), which is also similar to the expression patterns found for *MIR103A2* and *SOX4*. In summary, in the CT27 human stem cell model, the expression of *SOX4*, *PANK2*, and *MIR103A2* decreased following EVT differentiation, whereas in the CT29 human stem cell model, the expression of *SOX4*, *PANK2*, and *MIR103A2* increased following EVT differentiation.

Although gene expression patterns differed between the two hTSC lines, *SOX4*, *PANK2*, and *MIR103A2* showed consistent regulatory trends within each cell line, either increasing or decreasing together during EVT differentiation. These findings suggest a regulatory cascade in which *SOX4* modulates *PANK2* to control miR-103a-3p expression during EVT differentiation, ultimately regulating EVT invasion. These findings align with our previous in vitro findings in primary trophoblast cells, where *SOX4* silencing inhibited activin A-induced miR-103a-3p expression through *PANK2* regulation. We applied the data from GSE174481 and E-MTAB-6701 to confirm the above observations in vivo [33]. Prior to the analysis, the single-cell data were preprocessed as described by Shannon et al. [34]. Trophoblasts were then clustered by determining the subsets of cells expressing known trophoblast gene markers. As expected, this analysis provided eight clusters representing four CTB states (CTB1-4; TEAD4+, ELF5+, and EGFR+), one CTB state expressing SCT-associated genes termed the SCT precursor state (SCTp; ERVFRD-1+, CGA+), two column trophoblast states (cCTB1/2; NOTCH1+, ITGA5+), and one EVT state (EVT; HLA-G+, ITGA1+).

Using the Seurat package, the expression of *SOX4* was compared across all eight trophoblast clusters. Higher *SOX4* expression was detected in the EVT cluster. This finding is consistent with the trends observed in CT29 cells but not in CT27 cells (Fig. 5D). We next applied RT-qPCR to determine the expression profiles of the transcription factor *SOX4*, miR-103a-3p, and the host gene *PANK2* in CT29 cells on Day 0 (without differentiation induction), Day 6 of EVT differentiation (D6EVT), and Day 8 of EVT differentiation (D8EVT). The identity of EVTs was tested by performing immunofluorescence staining for the EVT lineage-specific marker HLA-G (Fig. S5D). The RT-qPCR results revealed the marked upregulation of *SOX4*, *PANK2*, and miR-103a-3p during hTSC-derived EVT differentiation (Fig. 5E). We then probed these three states with a SOX4 antibody. Compared with those on Day 0, the expression levels of the SOX4 protein increased significantly on Day 8 of EVT differentiation, as determined by a Western blot analysis (Fig. 5F). Using immunofluorescence staining, we detected differential SOX4 signals in CT29 stem cells, D6EVTs, and D8EVTs. In undifferentiated CT29 stem cells, the SOX4 signal was significantly weaker than that in D6EVTs or D8EVTs. From Day 6 to Day 8 of EVT differentiation, the SOX4 signal intensity gradually increased (Fig. 5G).

**Fig. 6:**
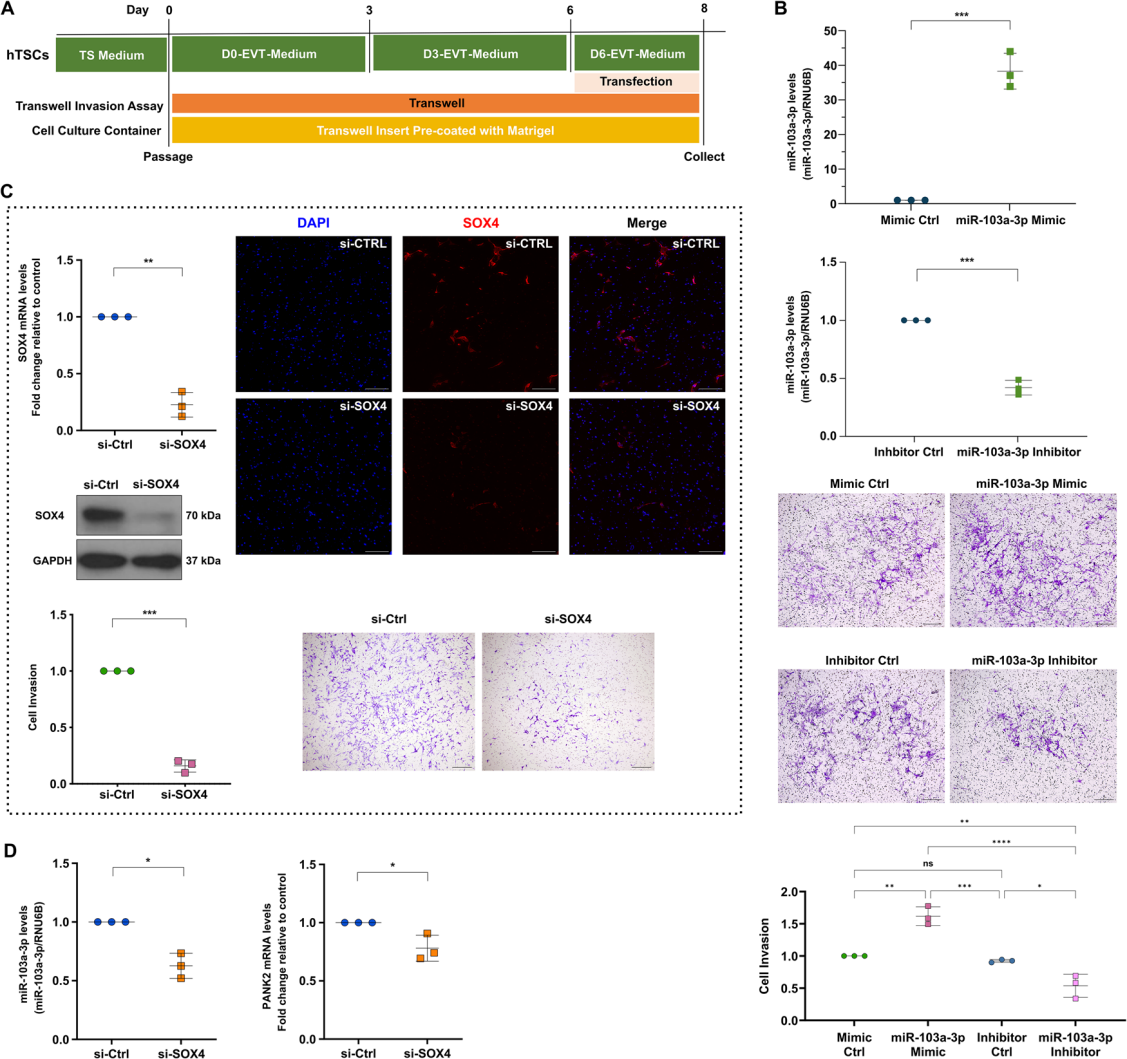
The transcription factor *SOX4* contributes to the increase in the expression of miR-103a-3p and its host gene *PANK2* during EVT differentiation and is required for the establishment of the normal invasive capacity of hTSC-derived EVTs. **A** Schematic diagram of the experimental procedures integrating transfection and Matrigel Transwell assays to study the roles of genes or miRNAs in cell invasion during the differentiation of hTSCs toward EVTs. **B** Trophoblasts were transfected on Day 6 of differentiation toward EVTs with the unspecific scrambled mimic control, miR-103a-3p mimic (5 nM), inhibitor control, or miR-103a-3p inhibitor (50 nM) for 48 h. The scatter plots show the efficiency of the forced expression and knockdown of miR-103a-3p, which were tested using RT-qPCR. The data were normalized to the endogenous control RNU6B. Statistical analyses of differences between two groups were performed using Student’s *t* test; significance was defined as *p* < 0.05. Each condition was performed in three independent experiments (*n* = 3). The representative images show the results of the Matrigel Transwell invasion assays and a summary of the quantitative results for invasion. Statistical analyses of differences between groups were performed using ANOVA and Tukey’s multiple comparisons test; significance *p* < 0.05. Each condition was performed in three independent experiments (*n* = 3). **C** Trophoblasts were transfected on Day 6 of differentiation toward EVTs with 50 nM scrambled siRNA (si-Ctrl) or si-SOX4 for 48 h. The efficiency of *SOX4* knockdown was examined by RT-qPCR and Western blotting. Representative images show the results of the Matrigel Transwell invasion assays. EVTs were transfected with or without the SOX4 siRNA on Day 6 and then collected and stained on Day 8. Brightfield images represent the staining of invaded cells with crystal violet. Immunofluorescence staining with a specific anti-SOX4 antibody and nuclear staining with DAPI are shown. The data were normalized to the control data. Statistical analyses of differences between two groups were performed using Student’s *t* test; significance *p* < 0.05. Each condition was performed in three independent experiments (*n* = 3). **D** The expression of miR-103a-3p and *PANK2* in the presence of a control siRNA or SOX4-targeting siRNA for 48 h was tested using RT-qPCR. The data were normalized to the levels of GAPDH (mRNA) or RNU6B (miRNA). Statistical analyses of differences between two groups were performed using Student’s *t* test; each condition was performed on three (*n* = 3) independent experiments. *p < 0.05; **p < 0.01; and ***p < 0.001. ns not significant. The data are presented as the means ± SDs. Scale bars: 100 μm (brightfield images); 200 μm (immunofluorescence staining).

Our initial findings indicate that *SOX4* regulates the miR-103a-3p-induced invasion of primary trophoblasts. We optimized a Matrigel Transwell culture system to study hTSC invasion at 48 h after siRNA knockdown, miRNA mimic transfection, or miRNA inhibitor transfection on Day 6 of EVT differentiation to investigate the role of *SOX4-*mediated miR-103a-3p in hTSC-derived EVT invasion (Fig. 6A). The results of the Matrigel Transwell assay showed that the forced expression of miR-103a-3p increased CT29 hTSC-derived EVT invasion, whereas the inhibition of miR-103a-3p significantly impaired the invasive ability of these EVTs on Day 8, indicating that miR-103a-3p may play a proinvasive role in regulating EVT function (Fig. 6B). We transfected CT29 hTSCs with a *SOX4*-targeting siRNA to investigate the regulatory role of *SOX4* on the expression of miR-103a-3p and its host gene *PANK2*, as well as its effect on EVT invasion. Following gene silencing, RT-qPCR and Western blotting were performed to assess the knockdown efficiency. We observed that the invasion of hTSC-derived EVTs on Day 8 was significantly inhibited after *SOX4* expression was silenced (Fig. 6C), and the expression of miR-103a-3p and its host gene *PANK2* was significantly decreased following *SOX4* knockdown (Fig. 6D). These data confirm that the regulation of miR-103a-3p by *SOX4* in human trophoblasts is achieved by targeting the host gene *PANK2*. In addition, the knockdown of *SOX4* expression in the late stage of EVT differentiation also decreased the expression of two iEVT markers (*SERPINE2* and *PLAC8*) and the EVT invasion marker *CSH1* (Fig. S5E) [35]. These observations indicate that *SOX4* and miR-103a-3p are essential for trophoblast invasion and the acquisition of the invasive capacity during EVT differentiation.

## Discussion

In this study, we applied small RNA-seq to first-trimester primary trophoblasts to comprehensively analyze the regulatory effects of activin A treatment on miRNA expression patterns in human trophoblast cells and to improve our understanding of the disruption of the expression of the growth factor activin A and miRNAs in the development of pregnancy disorders. Here, we identified 98 miRNAs whose expression was upregulated or downregulated following activin A treatment, with miR-103a-3p being the most important in the activin A-induced miRNA-mRNA regulatory network. We further elucidated the regulatory mechanisms used by activin A to promote the expression of miR-103a-3p and enhance primary trophoblast invasion through canonical SMAD2/3-SMAD4 signaling as well as the noncanonical *SOX4* induction of miR-103a-3p via *PANK2*. We identified that *SOX4*, miR-103a-3p and *PANK2* expression were concurrently induced during hTSC-derived EVT differentiation. Furthermore, the inhibition of *SOX4* and its downstream target miR-103a-3p attenuated the invasion of both primary trophoblasts and hTSC-derived EVTs. Consistent with these findings, *SOX4* silencing during late-stage EVT differentiation suppressed the expression of the EVT differentiation markers *SERPINE2*, *PLAC8* and *CSH1*. These observations demonstrate that the regulation of miR-103a-3p by *SOX4* is essential for trophoblast invasion and the acquisition of the invasive capacity during EVT differentiation. The miR-103a-3p gene (*MIR103A2*) is located within the introns of *PANK2* gene, which is a protein-coding gene. The intronic miRNA genes often exhibit co-regulated expression with host genes, resulting in synchronized expression patterns. This co-transcription typically responds to specific cellular signals or developmental cues [36]. However, certain intronic miRNAs may be independently regulated. Both miRNAs and their host genes can demonstrate tissue-specific expression, essential for regulating developmental processes and maintaining tissue homeostasis. This specificity is driven by distinct combinations of transcription factors, enhancers, and epigenetic marks in specific cell types [37]. Our findings reveal the co-expression pattern of miR-103a-3p and the host gene *PANK2*, as well as their relationship with transcription factor *SOX4*. Through the analysis of ChIP-Seq dataset GSE174602 [38], we identified a potential intermediate transcription factor, NF-Y (Nuclear Transcription Factor Y). The promoter region of the *NFYA* gene can be directly bound by SOX4 in human endometrial stromal cells (Fig. S6A). *NFYA* encodes the NF-YA subunit of NF-Y, a transcription factor complex recognized as a canonical transcriptional activator that binds to key regulatory elements known as CCAAT boxes across various tissues in humans [39]. This CCAAT box is located 58 to 100 nucleotides upstream of the transcriptional start site (TSS) of *PANK2*. Polster et al. also validated that the binding of NF-Y to the *PANK2* promoter has a significant impact on its promoter activity [40]. Additionally, we also found that the expression patterns of the *NFYA* gene in CT27 and CT29 hTSCs are consistent with those of *SOX4*, *PANK2*, and miR-103a-3p (Fig. S6B). But the deeper understanding of the regulatory mechanisms involved will require further investigation.

Previously, activin A was shown to be localized in CTBs and SCTs of the placenta across all gestational ages [41]. Furthermore, more recent studies using single-cell RNA-seq of the human maternal-placental interface have revealed abundant activin A expression in the placental STB and maternal decidua [33, 42]. During the first trimester and following decidualization, activin A expression is upregulated in decidualized stromal cells that are secreted into the intervillous space [43, 44]. Multiple studies have suggested that activin A promotes trophoblast invasion by regulating ECM-related genes such as *MMP2*, *MMP9*, *ITGB1*, and *CDH2*, suggesting a proinvasive role for activin A during early pregnancy and its involvement in trophoblast invasion-related pregnancy disorders[16, 45, 46]. However, recent single-cell placental transcriptome analyses showed low expression of *MMP9* and *CDH2* in EVTs, in contrast to earlier studies. This discrepancy may reflect the limitations of traditional trophoblast cell lines (HTR8/SVneo, JAR, and JEG-3), which differ substantially from trophoblasts in vivo [47]. We therefore used both primary human trophoblast and hTSC models to better recapitulate in vivo conditions.

We identified miR-103a-3p as the core regulator of the activin A-induced signaling network. miR-103a-3p belongs to the miRNA-103/107 family and can target the miRNA processing gene *Dicer* [48]. This finding may partially explain the key role of miR-103a-3p in activin A-induced miRNA-mRNA signaling, as it may influence the biosynthesis of other top miRNAs through a Dicer-dependent mechanism. The miRNA-103/107 family is also proposed to be involved in the induction of the EMT by downregulating the expression of miRNA families that target EMT-associated genes such as *EIF1AX*, *CALU*, and *TGFBR3*. Previous reports have shown that in the limbal epithelium, the miR-103/miR-107 family is instrumental in orchestrating multiple characteristics of epithelial stem cells by targeting Wnt signaling [49]. We also identified a particularly interesting gene, *AXIN2*, which was significantly inhibited by miR-103a-3p forced-expression. *AXIN2*, which is activated by β-catenin/T cell factor and is proposed to be involved in a negative feedback loop that helps regulate the duration and intensity of Wnt signaling [50]. Recently, a paper published by the Vento Tormo Lab found that EVT invasion is characterized by the inhibition of Wnt signaling, along with down-regulation of the Wnt target *AXIN2* [35]. Suggesting that miR-103a-3p may also be involved in the differentiation of trophoblast towards the extravillous pathway by modulating the Wnt signaling pathway. To our knowledge, the effect of the miR-103/107 family on human trophoblast invasion has not been reported, and our study, combined with primary and hTSC models, demonstrated that miR-103a-3p is involved in activin A-induced primary trophoblast invasion. However, a limitation is that the cell culture system of the hTSC model developed by Okae et al. contains the TGF-β pathway antagonist A83-01, thus preventing the validation of the effect of activin A [21]. Many studies have demonstrated that dynamic changes in TGF-β signaling are involved in trophoblast maintenance and differentiation in vivo [35]. Recent studies have attempted to modify hTSC culture conditions to investigate TGFβ signaling during EVT differentiation [51]. Future research can conduct relevant validations once these modified models are fully established. Our findings suggest that miR-103a-3p is preferentially expressed in CT29 hTSC-induced EVTs rather than in the stem cell state and that the disruption of miR-103a-3p reduces hTSC-derived EVT invasiveness during hTSC differentiation. These findings suggest that the dynamics of miR-103a-3p expression regulate the hTSC stemness-to-differentiation transition and trophoblast invasion. Activin A binds to ACVR type I receptors (*ALK4*/*ALK7*) and ACVR type II receptors, primarily triggering canonical SMAD2/3-SMAD4 signaling. Our results indicate that the inhibition of *SMAD2*, *SMAD3*, and *SMAD4* directly affects miR-103a-3p expression to varying degrees, with low expression of *SMAD4* significantly suppressing the expression of both the host gene *PANK2* and miR-103a-3p. Furthermore, we found that *SOX4*, which belongs to the SRY-related transcription factor family, acts as a new noncanonical target gene of activin A, participating in the regulation of activin A-induced *PANK2* and miR-103a-3p expression. The direct transcriptional targets of *SOX4* (*i.e*., *AGO1*, *RHA*/*DHX9*, and *DICER*) are important components of the RNA-induced silencing complex and miRNA processing [52]. A potential regulatory mechanism might involve the binding of SOX4 to the *DICER* promoter to induce the upregulation of multiple downstream miRNAs, including miR-103a-3p, at the transcriptional level. The subsequent increase in miR-103a-3p expression then downregulates *DICER* expression at the posttranslational level, thereby inhibiting the expression of related miRNAs and forming a negative feedback loop. This result is consistent with our finding that more miRNAs are downregulated following activin A treatment.

In the hTSC model, our data revealed that the expression patterns of *SOX4* and miR-103a-3p were conserved, with their expression increasing as CT29 cells differentiated into EVTs. Low *SOX4* expression inhibited the expression of the host gene *PANK2* and miR-103a-3p and prevented late-stage differentiated EVTs from acquiring invasive properties. Interestingly, the expression patterns of *SOX4* and miR-103a-3p in CT27 cells differed from those in CT29 cells, with higher expression levels in the stem cell state than in EVTs derived from CT27 cells. Sex-dependent gene expression has been observed in the placenta throughout gestation [53, 54]. Importantly, CT27 cells are female, whereas CT29 cells are male. Therefore, sex differences were considered and verified. When the data were split by sex, no significant sex differences in *SOX4* expression in different trophoblast clusters of the first-trimester placenta were observed (Fig. S7A). Surprisingly, when combined with data on the expression patterns of *SOX4* and miR-103a-3p in female CT30 cells, the results are similar to those we obtained in male CT29 cells (Fig. S7B). The differences in the expression of the *SOX4* mRNA and miR-103a-3p in different TSCs are more likely related to individual differences in the samples from which the cell lines were derived rather than to the sex of the tissue. Research has also revealed differences among various hTSC lines, with CT29-derived organoids in hTSC 3D culture showing the fastest capacity for EVT differentiation, followed by CT27- and CT30-derived organoids [34]. Therefore, we suggest that researchers consider both sex differences and individual variations when using TSCs as the experimental model in future studies.

At the cellular level, we identified proinvasive roles for activin A and miR-103a-3p in trophoblast invasion. Furthermore, through the analysis of public datasets, we found that among the TGF-β family members reported to be associated with preeclampsia, the increased levels of activin A in villous tissue and decidua basalis tissues of preeclampsia patients were significantly greater than those of other growth factors (Fig. S8A) [7]. Interestingly, a proteomic analysis of blood samples from preeclampsia patients at 22–28 weeks of gestation revealed significantly higher levels of the activin A protein than those in the control group (Fig. S8B) [55]. Additionally, the expression of exosomal miR-103a-3p was increased in the plasma of preeclampsia patients during early (11–14 weeks), middle (22–28 weeks), and late (32–38 weeks) gestation (Fig. S8C) [56], whereas the expression of nonexosomal miR-103a-3p was not different (Fig. S8D). These findings suggest that exosomes help bridge communication between fetal and maternal cells by transporting miRNAs. Moreover, the period of 22–28 weeks of gestation is likely a window for the early diagnosis of preeclampsia, during which the expression of activin A and exosomal miR-103a-3p significantly increases. However, more clinical research and the creation of statistical diagnostic models are needed to identify aberrant expression levels of the activin A protein and miR-103a-3p in the blood for an early diagnosis.

Overall, our study utilized small RNA sequencing technology combined with primary trophoblast and hTSC models to elucidate the mechanism by which activin A regulates miRNA expression in the human placenta. We identified the roles of and a novel interaction between the transcription factor *SOX4* and miR-103a-3p, leading to the promotion of trophoblast invasion. Furthermore, we identified the possibility that *SOX4*-mediated modulation of miR-103a-3p is essential for extravillous trophoblast differentiation. Future research should focus on the molecular mechanisms underlying the *SOX4*-induced differentiation of human trophoblast stem cells toward EVTs. Importantly, our analysis of public transcriptomic and proteomic datasets revealed that elevated activin A and exosomal miR-103a-3p expression during mid-gestation could serve as potential biomarkers for the early diagnosis of preeclampsia. Future studies are needed to model relevant biomarker trajectories and determine the sensitivity and specificity of activin A and miR-103a-3p in preeclampsia.

## Supplementary information

Supplemental Figure Legends

Fig.S1

Fig.S2

Table S1

Table S2

Table S3

Fig.S3

Fig.S4

Fig.S5

Fig.S6

Fig.S7

Fig.S8

Original Western Blot Image

## Data Availability

The data are available upon reasonable request. The raw small RNA sequencing data reported in this paper have been deposited in the Genome Sequence Archive for Human (GSA-Human) under accession No. HRA008074 and are available upon reasonable request.
