# Supplementary material for: Elucidating the roles of microRNA-103a-3p in trophoblast invasion and SOX4-mediated extravillous differentiation induced by activin A
Source: Cell Death Dis. 2026 Apr 10;17(1):466. doi: 10.1038/s41419-026-08665-6 (PMC13181086; doi:10.1038/s41419-026-08665-6)
Supplement: Supplementary file 1 — Supplemental Figure Legends [file 41419_2026_8665_MOESM1_ESM.docx]

**Supplemental Figure Legends**

**Supplemental Figure S1.** **Characterization of first-trimester human primary trophoblasts**

Image of immunofluorescence staining for KRT7 and HLA-G in human primary trophoblasts. Nuclei were stained with DAPI. Scale bar: 200 μm.

**Supplemental Figure S2.** **Summary of differentially expressed miRNAs in human primary trophoblasts treated with or without activin A**

(A) Heatmap of the hierarchical clustering analysis showing a total of 98 miRNAs that were differentially expressed according to the small RNA sequencing analysis (Q value ≤ 0.001, absolute value of Log 2 (fold change) ≥ 1) of control and 50 ng/mL activin A-treated human primary trophoblasts (Row Z score). (B) GO analysis of activin A-induced differentially expressed miRNAs highlighting the top 10 enriched terms for biological processes (BPs), cellular components (CCs), and molecular functions (MFs) in primary trophoblasts. (C) KEGG pathways enriched in the target genes of activin A-induced miRNAs in primary trophoblasts. The X-axis represents the number of genes annotated to a specific KEGG pathway, and the Y-axis represents the pathways.

**Supplemental Figure S3.** **Detection of miR-103a-3p predicted target genes**

(A) RT-qPCR validation of the expression of miR-103a-3p predicted target genes *ANKFY1*, *CA12*, *NUFIP2*, and *PRKAR2A* in primary trophoblasts transfected with the unspecific scramble mimic control or 5 nM miR-103a-3p mimic for 48 h. Statistical analyses between two groups were performed using Student’s t test: significance P<0.05. Each condition was performed on five (n=5) independent experiments. (B) The left panel represents the pathway enrichment analysis based on the top 20 integrated score target genes of miR-103a-3p generated from mirDIP. The right panel shows the target genes mapped to the enriched pathways identified in the left panel. (C) RT-qPCR validation of the expression of miR-103a-3p predicted target genes *AXIN2* in primary trophoblasts transfected with the unspecific scramble mimic control or 5 nM miR-103a-3p mimic for 48 h. (D) RT-qPCR validation of the expression of miR-103a-3p predicted target genes *EIF1AX*, *CALU*, and *TGFB3* in primary trophoblasts transfected with the unspecific scramble mimic control or 5 nM miR-103a-3p mimic for 48 h. Statistical analyses between two groups were performed using Student’s t test; significance P<0.05. Each condition was performed on five (n=5) independent experiments. *P<0.05; **P<0.01; ***P<0.001. Data are mean ± SD.

**Supplemental Figure S4. Analysis of the potential interactions between the transcription factor *SOX4*, the host gene *PANK2*, and activin A using publicly available datasets**

(A) Expression levels of PANK2 obtained from the RNA-seq analysis of human mammary epithelial cells (HMLEs) transduced with lentiviral vectors containing expression cassettes for either a tamoxifen-inducible partial estrogen receptor (ER) or a fusion of the ER with SOX4 (ER-SOX4). The data were generated from the RNA-seq dataset GSE125642 (grouped by ERmin, ERplus, ERSOX4min, and ERSOX4plus; n=2). The expression levels are presented as the means ± SDs of TPM-normalized expression. ERmin, ER minus 4OH-tamoxifen; ERplus, ER plus 4OH-tamoxifen; ERSOX4min, ERSOX4 fusion minus 4OH-tamoxifen; ERSOX4plus, ERSOX4 fusion plus 4OH-tamoxifen. (B) The expression levels of PANK2 in human breast cancer cell lines treated with either an siRNA targeting SOX4 or a nontargeting control siRNA. The data was generated from the RNA-seq dataset GSE158295 (N=2). The expression levels are presented as TPM-normalized expression values, means ± SDs. (C) Bar graph showing the expression levels of SOX4 in time series experiments in which 10 ng/mL activin A was added to human embryonic stem cell (hESC) cultures. The data were generated from the RNA-seq dataset GSE111717 (control group (0, 12 h) and activin A treatment group (2.5 h, 4 h, 12 h); n=2). The mean values are presented as normalized counts.

**Supplemental Figure S5.** **The expression patterns of SOX4, PANK2, and miRNA103a-3p in the different states of CT27**

(A) Box plot showing the expression levels of the *MIR103A1* and *MIR103A2* transcripts in the CT27 stem cell state and EVT state on Day 8 of differentiation (EVT27). (B) Box plot showing the expression levels of *SOX4* transcript variants in the CT27 stem cell state and EVT27. (C) Box plots showing the expression levels of four *PANK2* transcript variants related to *MIR103A2* transcription identified in Fig. 2C from the UCSC genome browser (left panel) and the expression levels of all *PANK2* transcript variants in the CT27 stem cell state and EVT27 state (right panel). The data was generated from the RNA-seq dataset GSE204722 (A-C). The expression levels are presented as TPM-normalized expression levels, means ± SDs (n=3). EVT27, EVT cells derived from CT27 cells, were collected on Day 8 of differentiation. (D) Images of immunofluorescence staining of CT29 hTSCs on Days 0, 6, and 8 of differentiation toward EVTs with specific HLA-G antibodies, and the nuclei were stained with DAPI. Scale bars: 200 μm. (E) CT29 hTSCs were transfected with the SOX4 siRNA on Day 6 to promote EVT differentiation. Representative data showing the mRNA expression levels of the *SERPINE2*, *PLAC8*, and *CSH1* were analyzed via RT-qPCR on Day 8 of EVT differentiation. Statistical analyses of differences between two groups were performed using Student’s t test; P< 0.05. Each condition was performed in three independent experiments (n=3). The data are presented as the means ± SDs. *P<0.05; **P<0.01; and ***P<0.001. ns, not significant.

**Supplemental Figure S6.** **Visualization of SOX4 Binding Enrichment and Peak Calling at the *NFYA* Gene Locus**

(A) The upper panel displays a representative genome browser view illustrating ChIP-Seq enrichment profiles for SOX4 at the *NFYA* gene region in human endometrial stromal cells. The lower panel displays the average signal density of SOX4 ChIP-Seq (red) and the input control (blue) across the *NFYA* gene body and its flanking regions. The x-axis represents genomic coordinates relative to the *NFYA* locus, encompassing 3 kb upstream of the TSS. The y-axis indicates normalized read density, expressed in Reads Per Kilobase per Million mapped reads, calculated using a 10 bp bin size. The prominent peak in the SOX4 track, relative to the input control, signifies a localized protein-DNA interaction within the *NFYA* regulatory region. Data were derived from the ChIP-Seq dataset GSE174602 (HA-SOX4 n=1; input control n=1). Trim Galore was utilized to filter the paired-end ChIP-Seq data, applying quality and adapter removal criteria. Reads shorter than 20 bp were excluded prior to alignment with hg38. Peak calling and genomic feature annotations were performed using HOMER. (B) The box plots illustrate the expression levels of *NFYA* transcript in the CT27 stem cell state and EVT27. The data was obtained from the RNA-seq dataset GSE204722. Expression levels are presented as TPM-normalized values, along with means and standard deviations (SDs, n=3). EVT27 represents EVT cells derived from CT27 cells, collected on Day 8 of differentiation.

**Supplemental Figure S7.** **Differential Expression of SOX4 in First-Trimester Trophoblast Clusters and CT30 During EVT Differentiation**

(A) UMAP and violin plots showing the *SOX4* expression levels across integrated first-trimester trophoblast clusters from female (n=6) or male (n=5) placentas. The data were generated from the integrated single-cell RNA-seq dataset GSE174481 (placentas, n=7) and the public repository ArrayExpress E-MTAB-6701 (placentas, n=4; decidua, n=4). The cell clusters were labeled according to the pipeline provided by the authors. (B) Representative mRNA or miRNA expression levels of SOX4, PANK2, and miR-103a-3p in CT30 hTSCs on Days 0, 6, and 8 of differentiation toward EVTs analyzed by RT-qPCR and normalized to those of GAPDH (mRNA) or RNU6B (miRNA). Statistical analyses of differences between groups were performed using ANOVA and Tukey’s multiple comparisons test; P< 0.05. Each condition was performed in three independent experiments (n=3). The data are presented as the means ± SDs. *P<0.05; **P<0.01; and ***P<0.001. ns, not significant.

**Supplemental Figure S8. Analysis of INHBA and miR-103a-3p expression in individuals with preeclampsia**

(A) The left box plots show the TPM expression levels of twelve TGF-β superfamily genes in villus and decidua basalis tissues from control subjects with an uncomplicated term birth and patients with early-onset preeclampsia. The right box plots show the statistical analysis of the data for INHBA in villus tissue or decidua basalis from 5 groups (uncomplicated TB, non-FGR EOPE, FGR, EOPE with FGR, and spontaneous idiopathic PTB without FGR). The data were generated from the RNA-seq dataset GSE203507 performed on paired villus tissues and decidua basalis, grouped by uncomplicated TB (VT, n=5; DB, n=5), non-FGR EOPE (VT, n=8; DB, n=8), FGR (VT, n=5; DB, n=5), EOPE with FGR (VT, n=7; DB, n=4), and spontaneous idiopathic PTB without FGR (VT, n=5; DB, n=5). The normalized expression data are presented as the means ± SDs of Log2 (count+1) values. A statistical analysis was performed on the raw data using the Wilcoxon test for multiple groups, the p.adj value for multiple comparisons was determined using the Benjamini‒Hochberg procedure, and p.adj<0.05 was considered significant and is indicated. *p.adj<0.05; **p.adj<0.01; ***p.adj<0.001; and ****p.adj<0.0001. VT, villus tissue; DB, basal plate decidua basalis; TB, uncomplicated term birth; EOPE, early-onset preeclampsia without fetal growth restriction; FGR, normotensive, nonanomalous preterm fetal growth restriction; EOPE plus FGR: EOPE with FGR; iPTB, spontaneous idiopathic preterm birth without FGR. (B) Box plot showing the maternal plasma levels of activin A in healthy (n=32) and EOPE (n=32) patients between 22 and 28 weeks. The data were generated from a longitudinal proteomic study (PMC6548389). The data are presented as the means ± SDs of the relative fluorescence units, and significance was inferred via Student’s t test. ****P<0.0001. (C) Heatmap showing the top 10 differentially expressed miRNAs in exosomes isolated from the plasma of PE patients. The data were generated from GSE94721, which included the top 10 differentially expressed miRNAs based on the statistical analysis of reads performed using DESeq2 (p.adj<0.01). The color scale represents the normalized miRNA expression. NP, normal pregnancy; PE, preeclampsia; 1st: exosomes isolated from maternal plasma during early gestation (*i.e.*, 11 to 14 weeks); 2nd, mid-gestation (*i.e.*, 22 to 28 weeks); 3rd, late gestation (*i.e.*, 32 to 38 weeks). (D) Box plot showing the expression level of miR103a-3p in total RNA collected from the blood samples of nondiseased control healthy (n=7), EOPE (n=5), and LOPE (n=5) patients. The data generated from GSE234611 are presented as the means ± SDs of Log2 (RKM+1), and the statistical analysis was performed with DESeq2. An adjusted p value<0.01 was considered significant and is marked. LOPE, late-onset PE.
