# Supplementary figures and images for: Elucidating the roles of microRNA-103a-3p in trophoblast invasion and SOX4-mediated extravillous differentiation induced by activin A
Source: Cell Death Dis. 2026 Apr 10;17(1):466. doi: 10.1038/s41419-026-08665-6 (PMC13181086; doi:10.1038/s41419-026-08665-6)

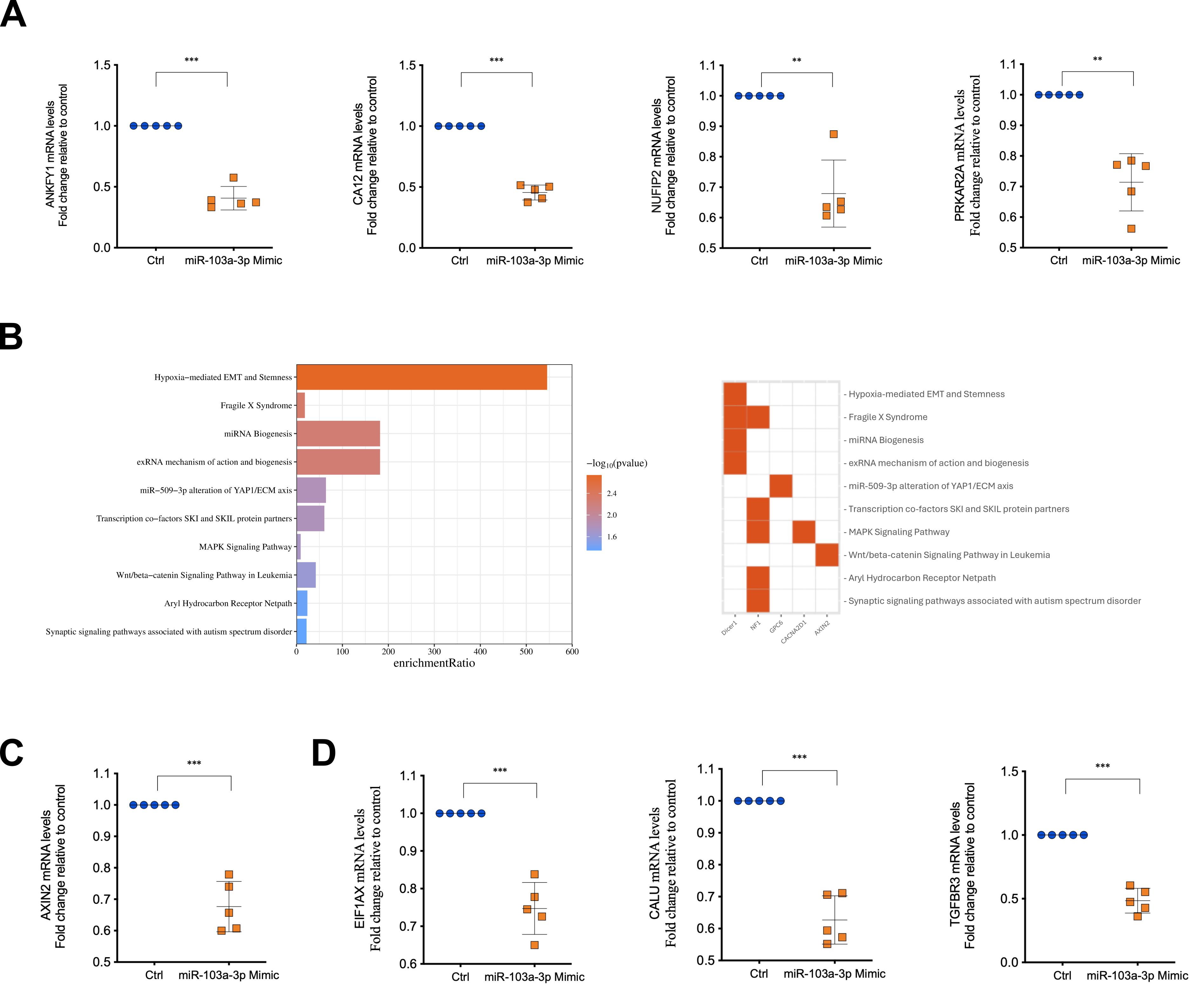

Supplement: Supplementary file 7 — Fig.S3 [file 41419_2026_8665_MOESM7_ESM.tif]

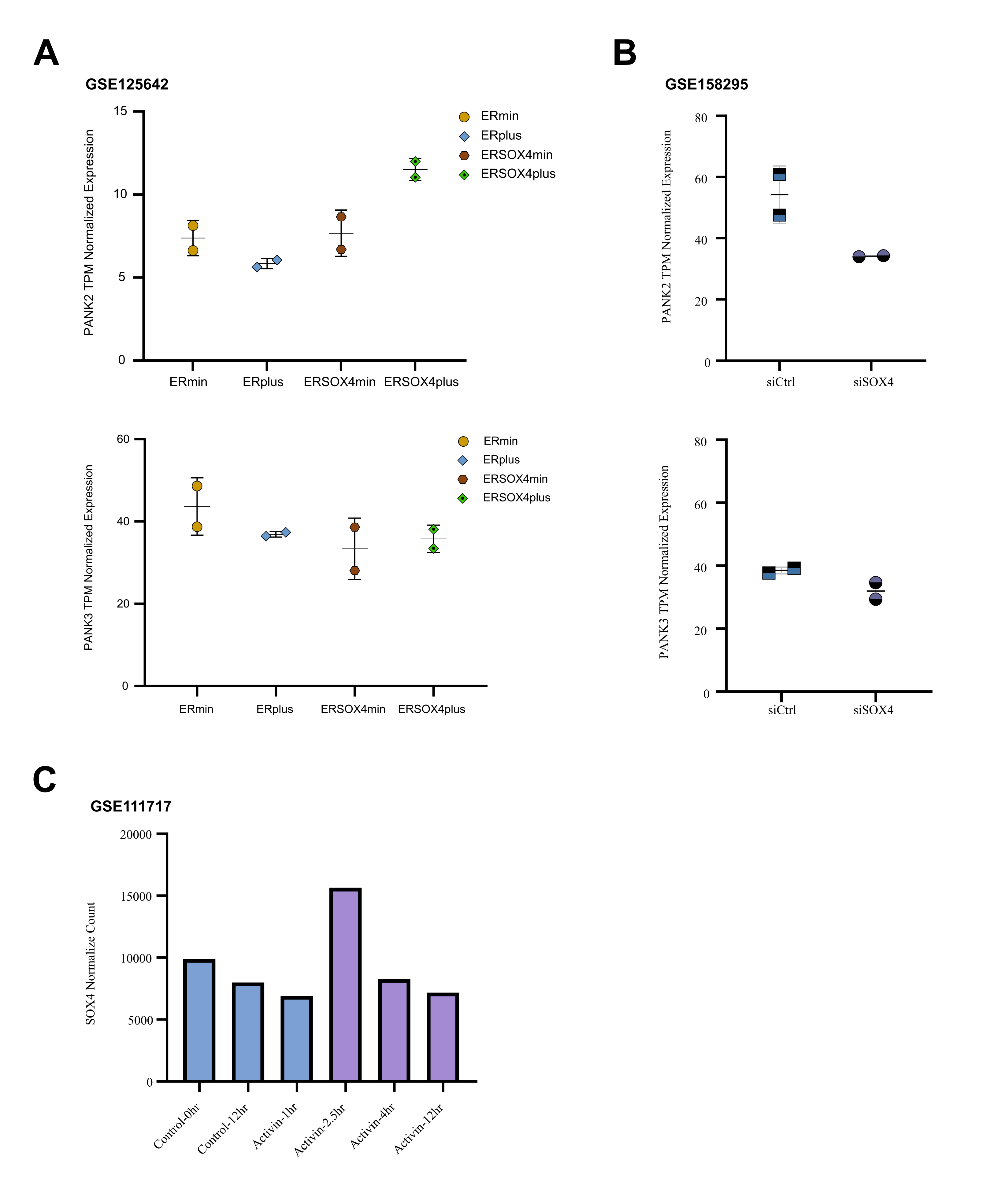

Supplement: Supplementary file 8 — Fig.S4 [file 41419_2026_8665_MOESM8_ESM.tif]

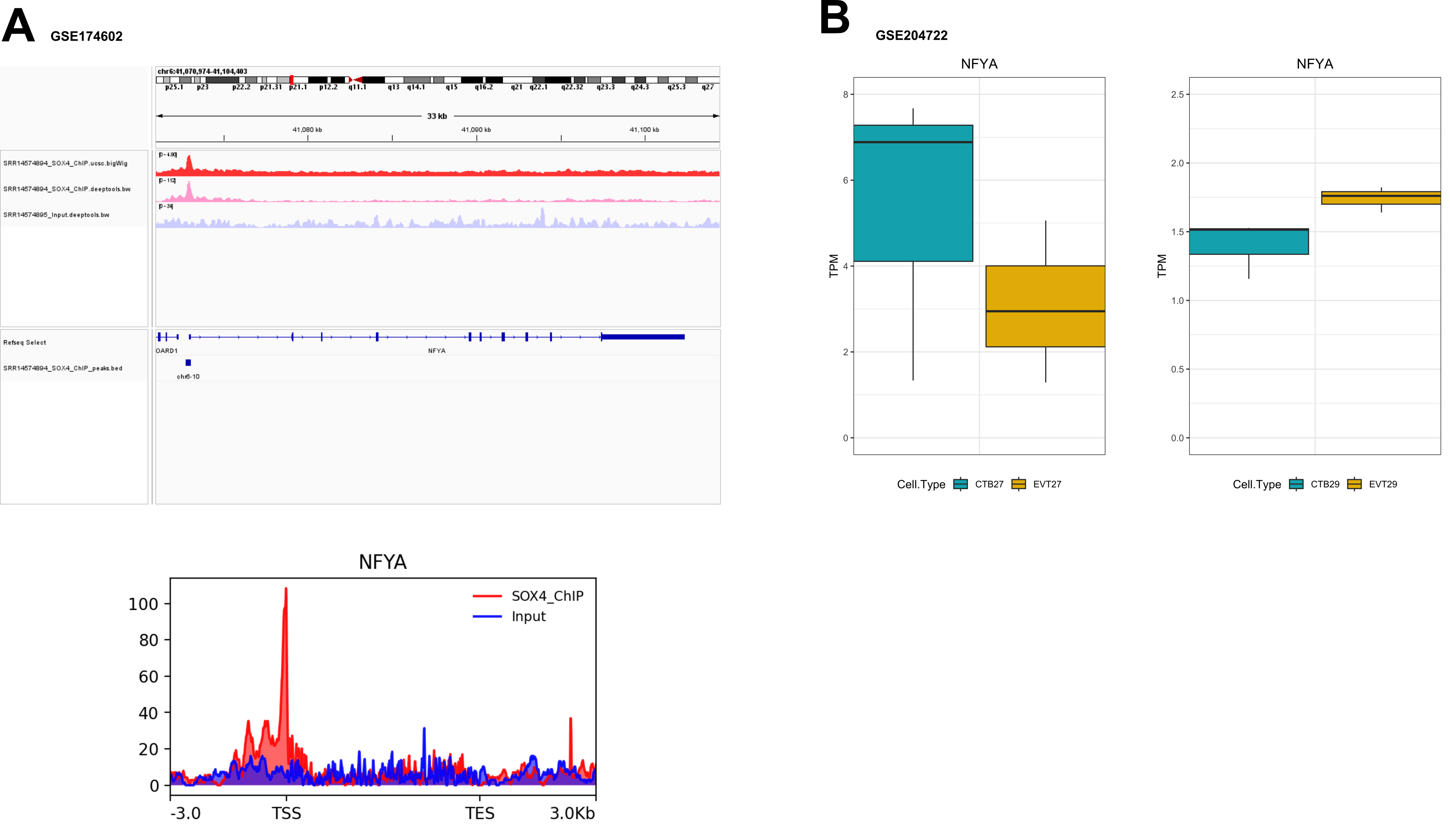

Supplement: Supplementary file 10 — Fig.S6 [file 41419_2026_8665_MOESM10_ESM.tif]
