# Supplementary material for: Elucidating the roles of microRNA-103a-3p in trophoblast invasion and SOX4-mediated extravillous differentiation induced by activin A
Source: Cell Death Dis. 2026 Apr 10;17(1):466. doi: 10.1038/s41419-026-08665-6 (PMC13181086; doi:10.1038/s41419-026-08665-6)

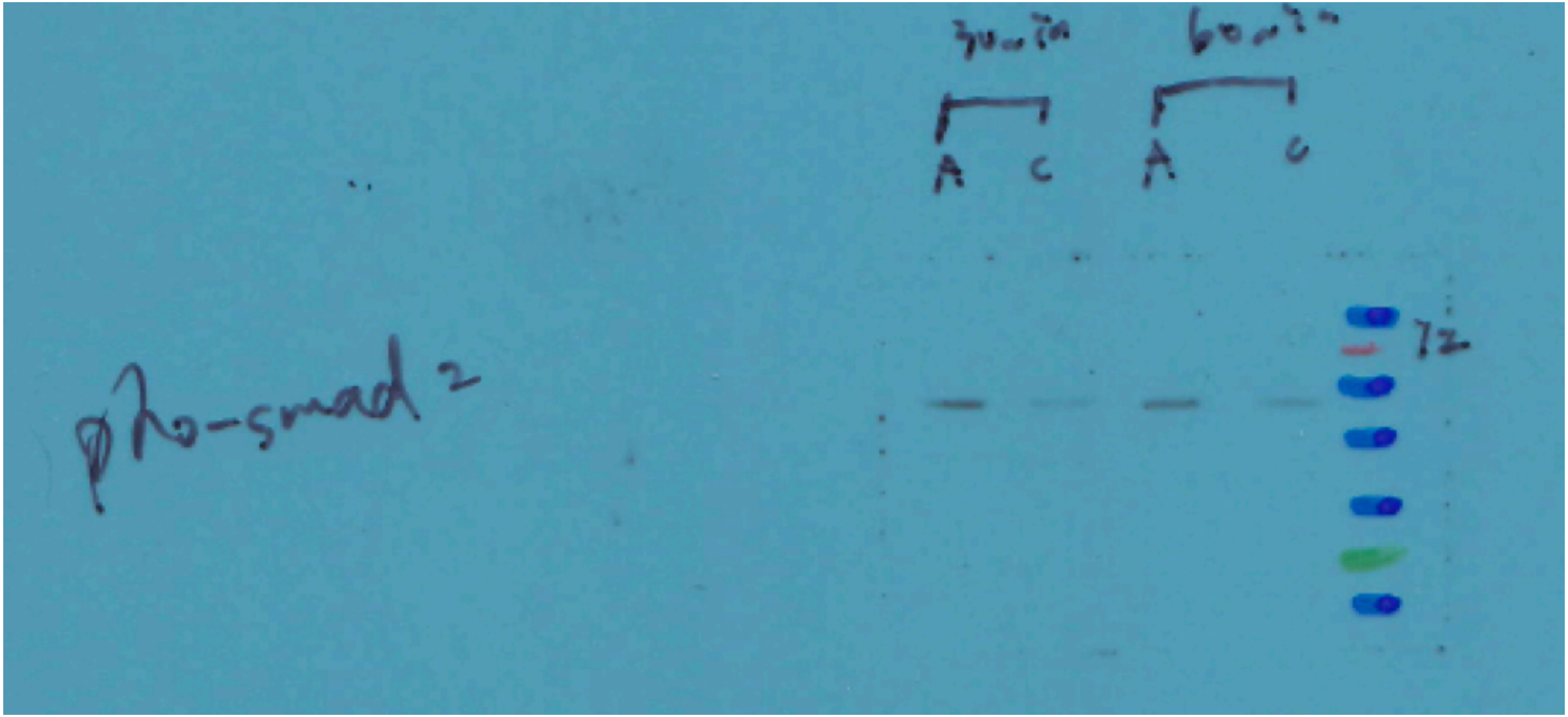
30min
60min
A
C
A
C
72
pho-smad 2

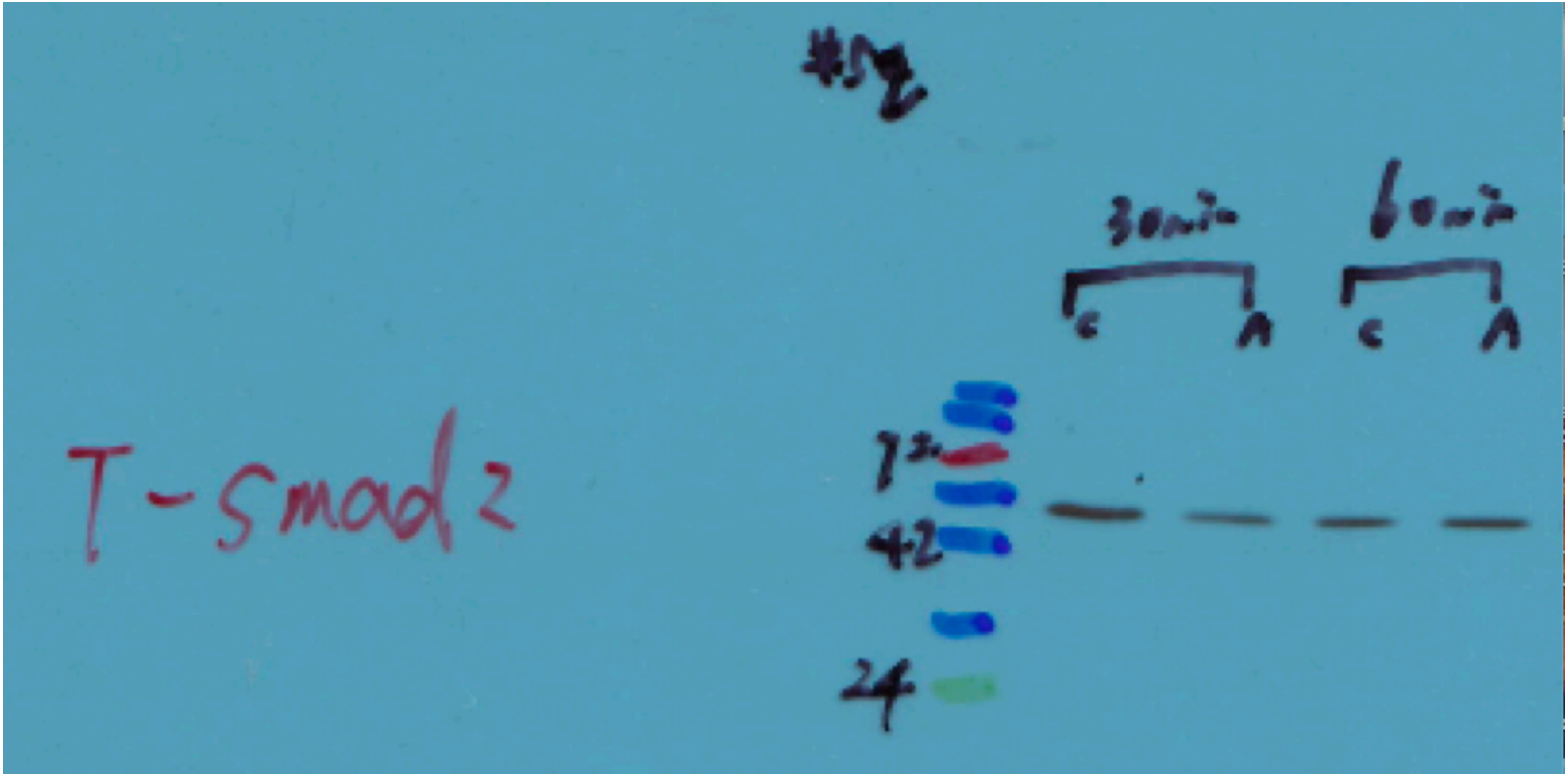
T-Smad2
30min
60min
C
A
C
A
42
24

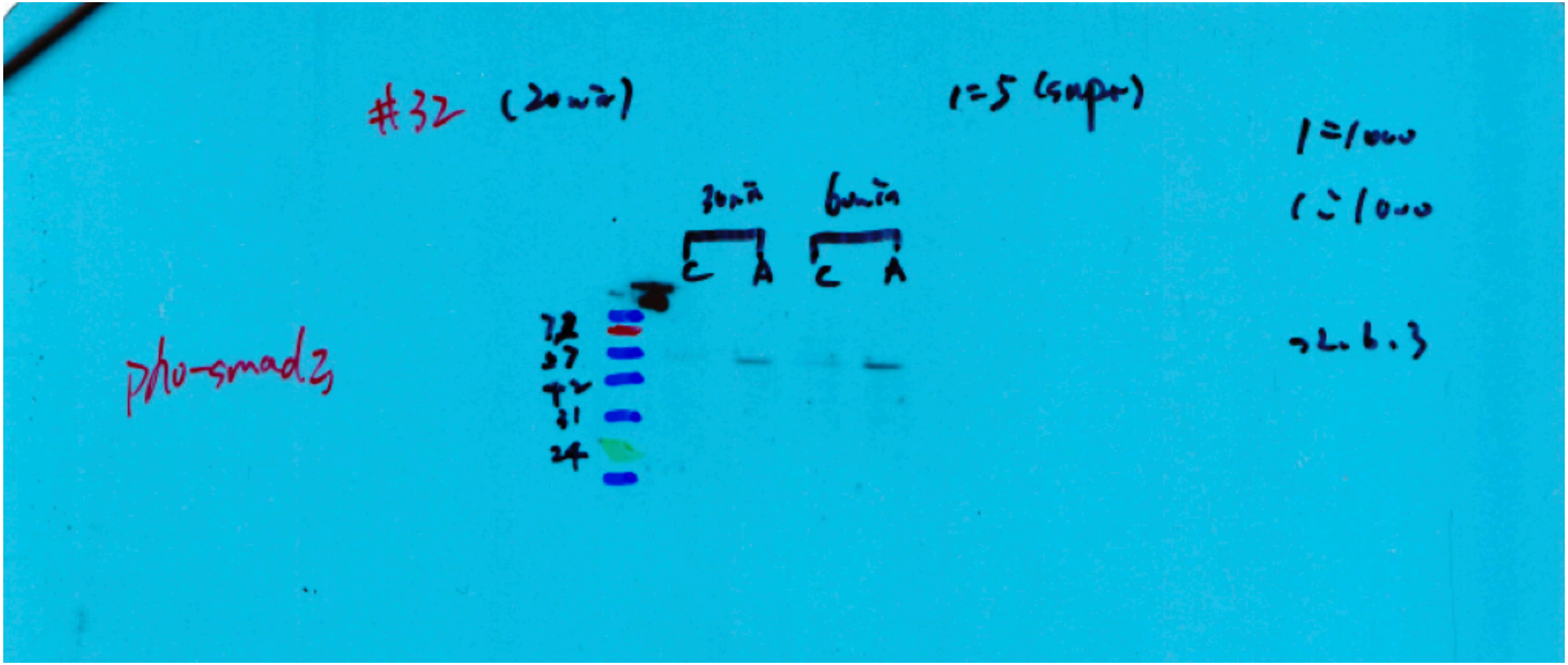
#32
1:5 (super)
30min
60min
C A C A
pho-smad3
72
57
42
31
24

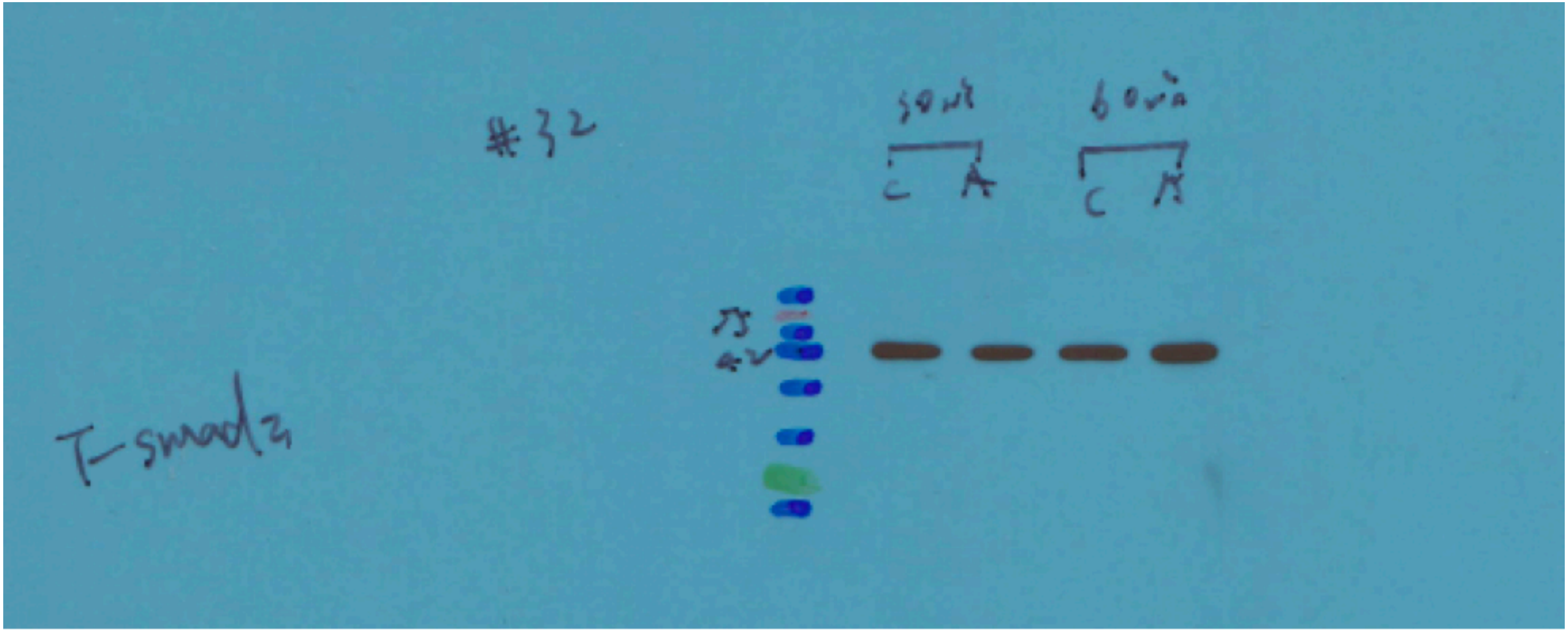

#32
C
A
C
75
T-smad2

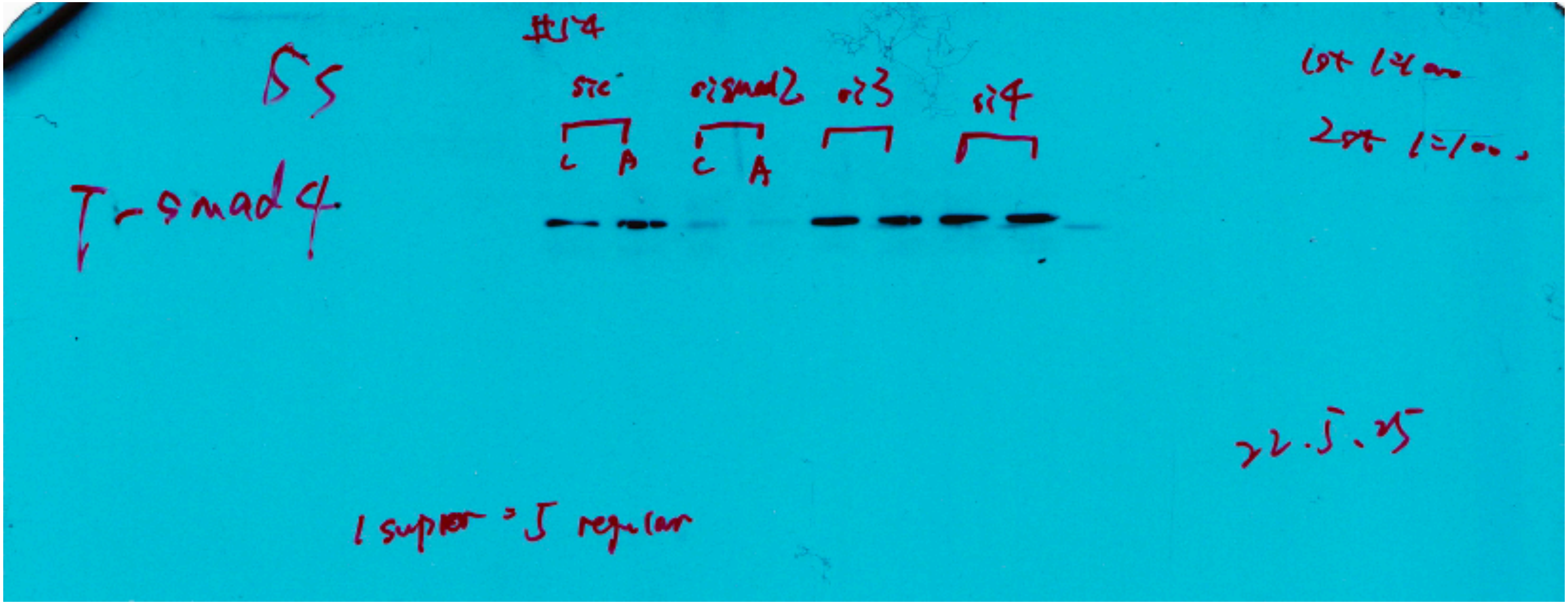
55
T-smad4
sic
sismad2
si3
si4
C
A
C
A
1 super = 5 regular
22.5.25

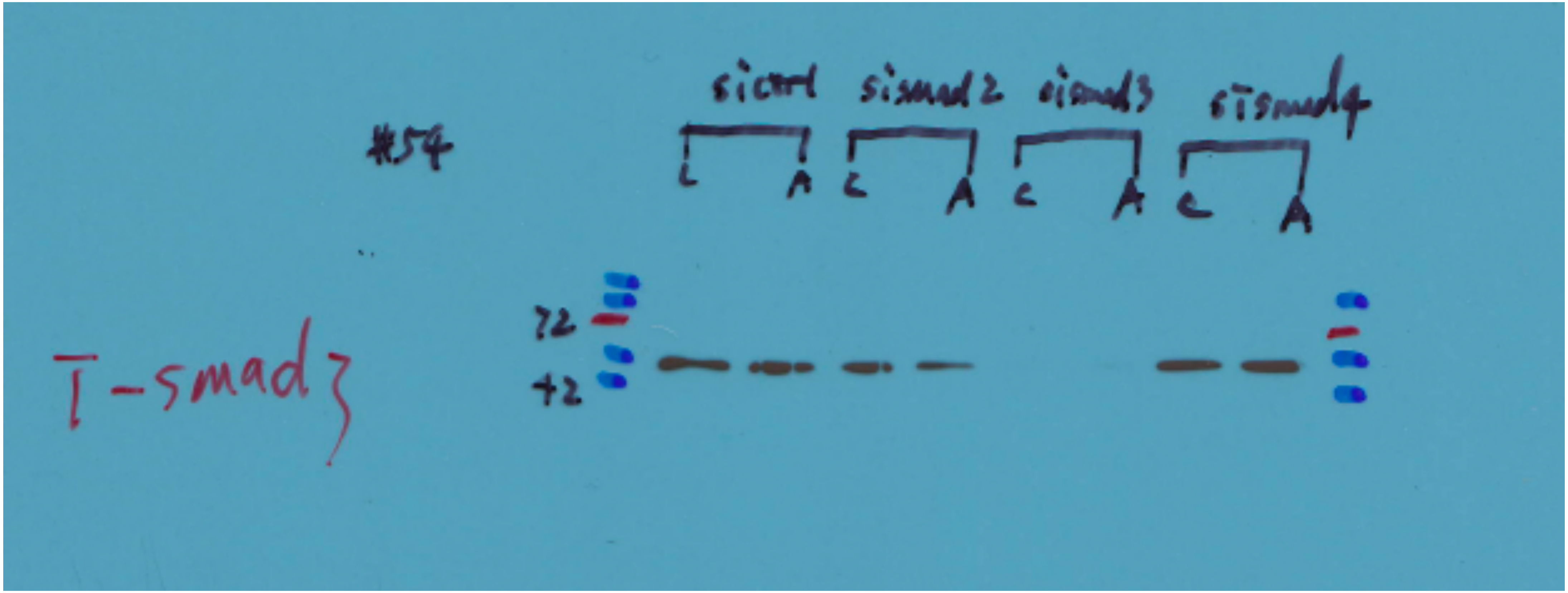
#54
sictrl
sismad2
sismad3
sismad4
C A C A C A C A
72
42
T-smad3

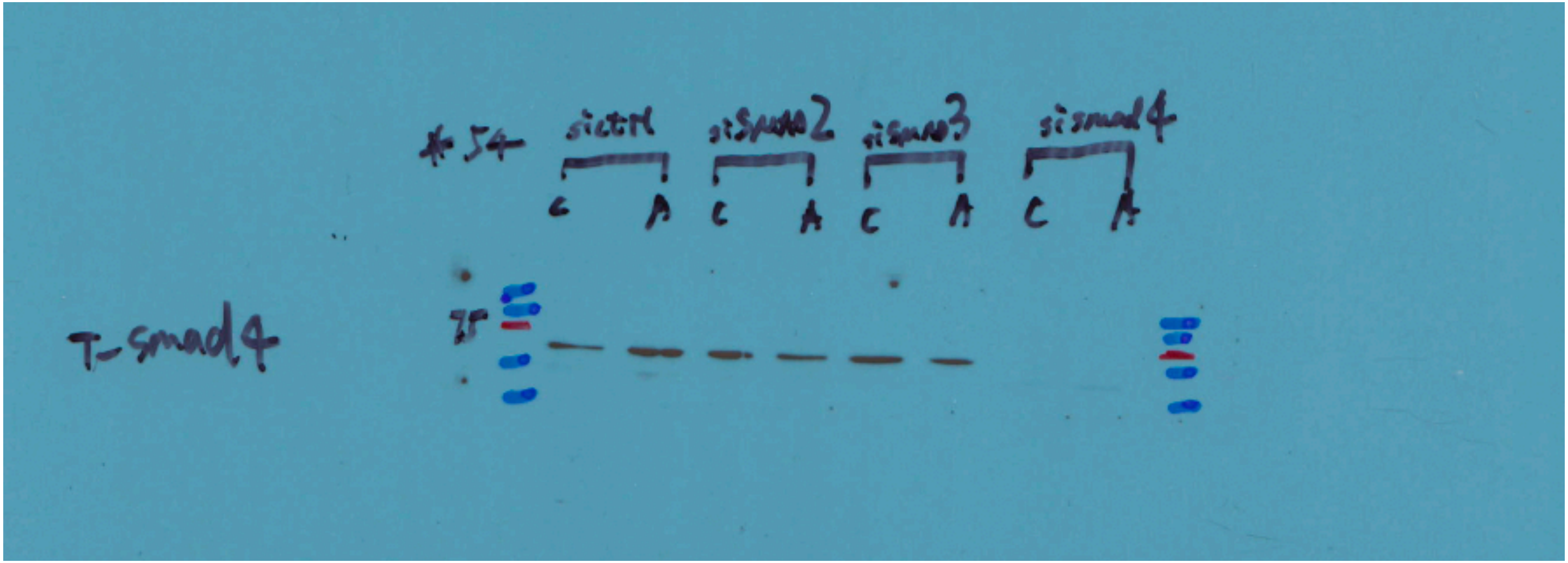
#54
sictrl
siSmad2
siSmad3
sismad4
C
A
C
A
C
A
C
A
75
T-smad4

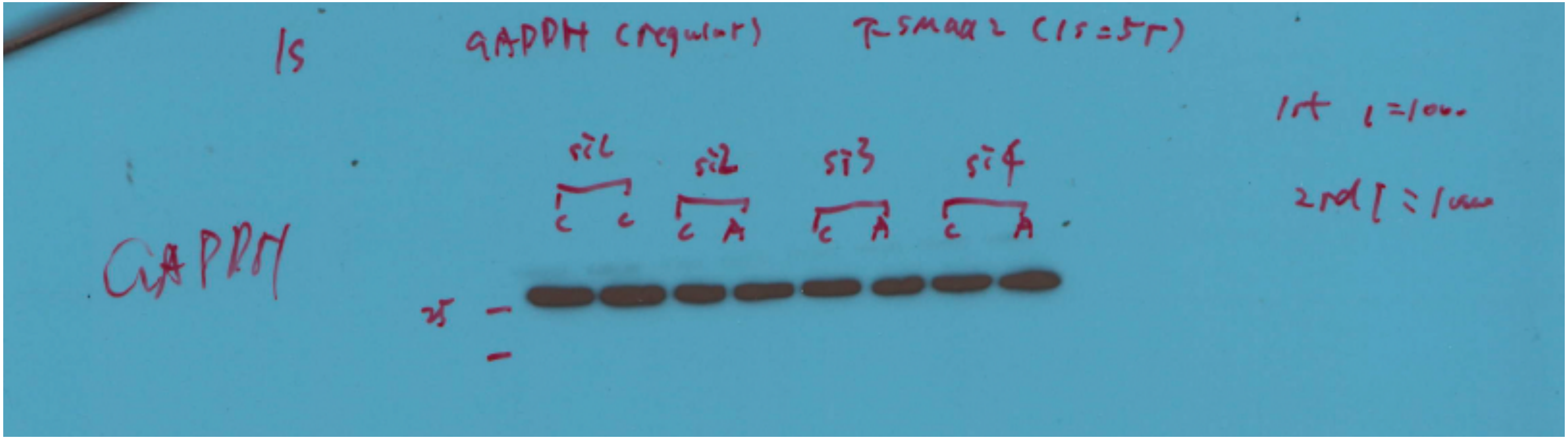
15
GAPDH (regular)
si1
si2
si3
si4
c c
c A
c A
c A
GAPDH
25
1st 1:1000
2nd 1:1000

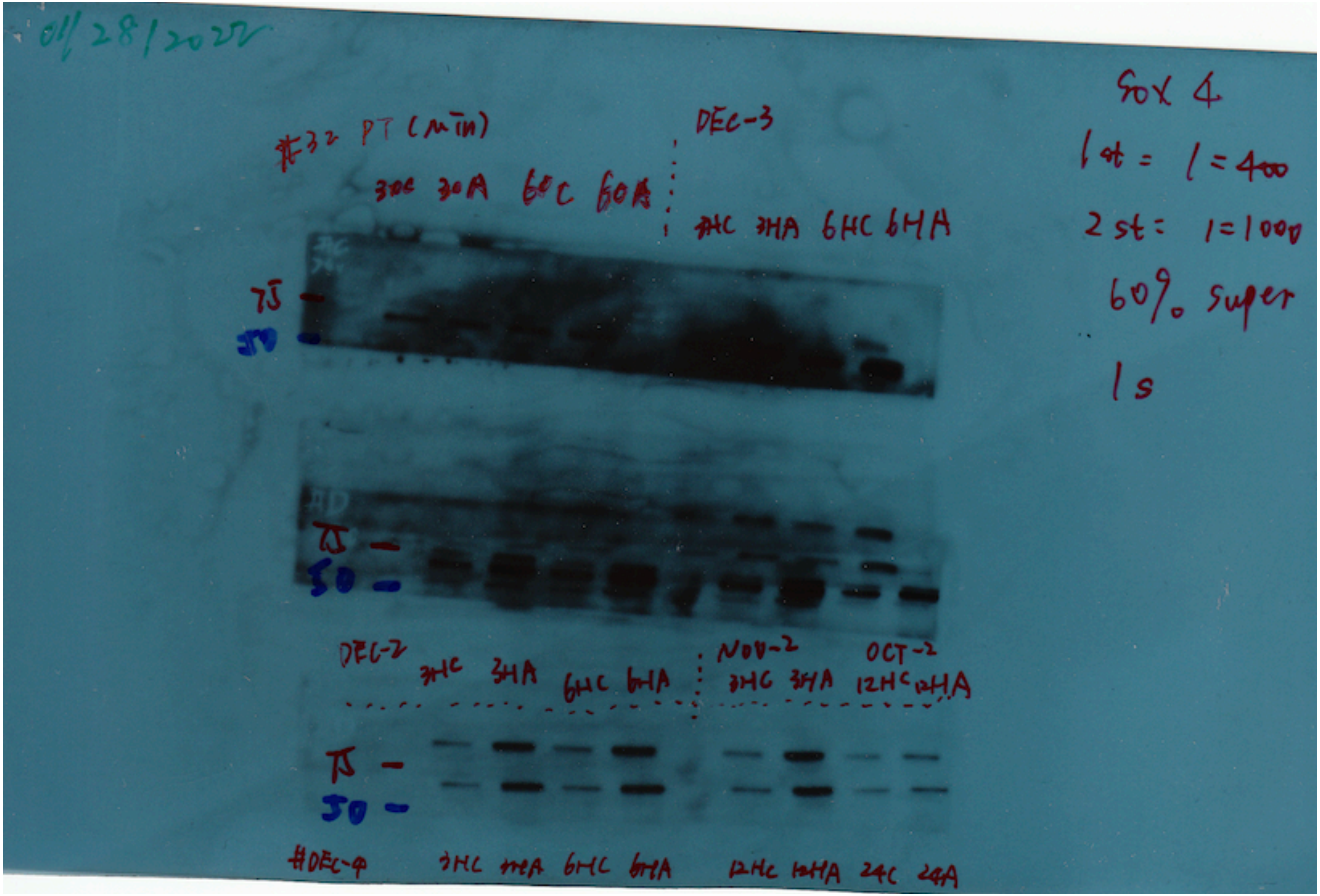
01/28/2022
Sox 4
1st = 1:400
2st: 1:1000
60% super
1s
#32 PT (min)
30C 30A 60C 60A
DEC-3
3HC 3HA 6HC 6HA
75
50
DEC-2
3HC 3HA 6HC 6HA
NOV-2
3HC 3HA
OCT-2
12HC 12HA
#DEC-4
3HC 3HA 6HC 6HA 12HC 12HA 24C 24A

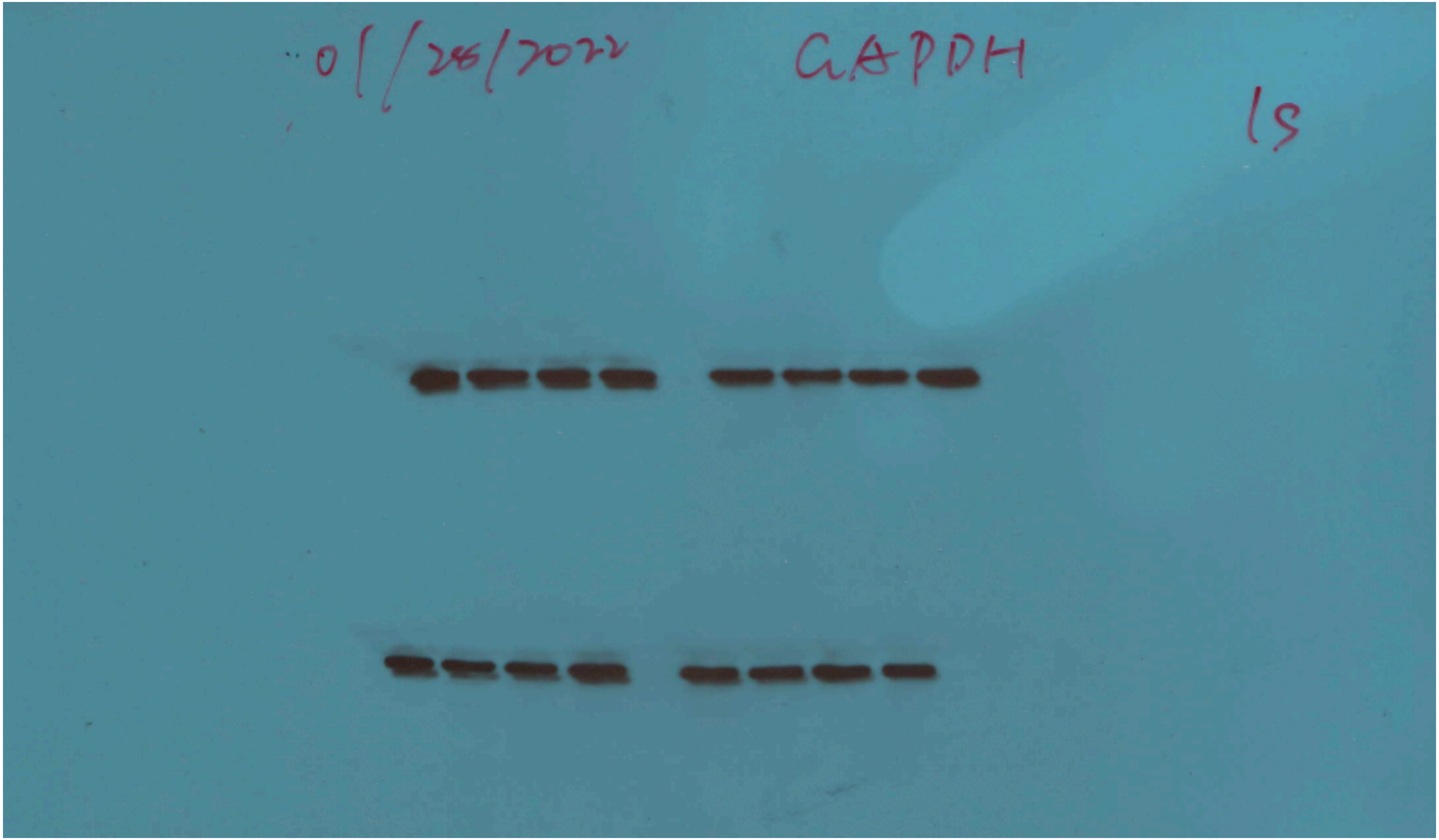
01/28/2022
GAPDH
15

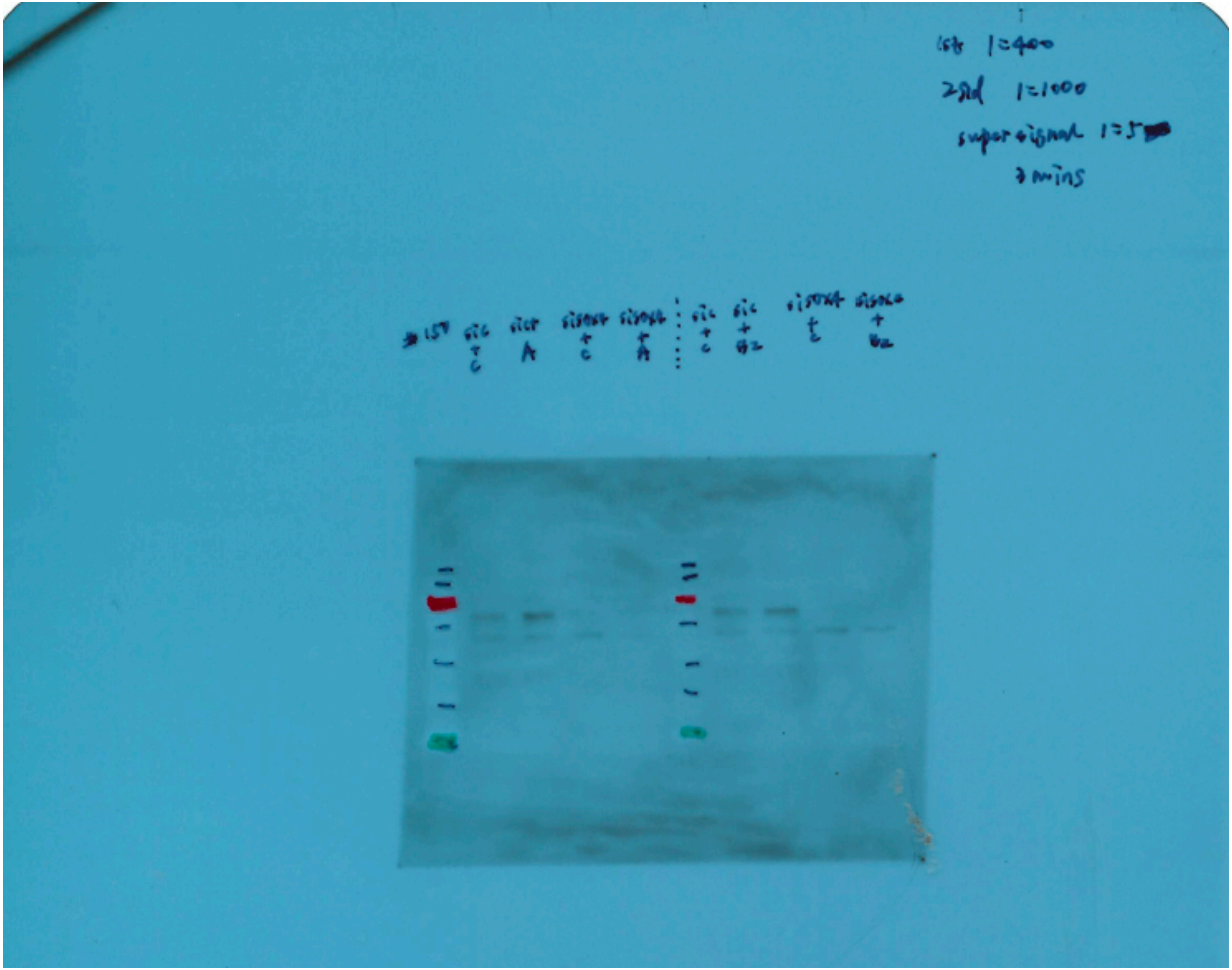
1st 1:400
2nd 1:1000
super signal 1:5[redacted]
3 mins

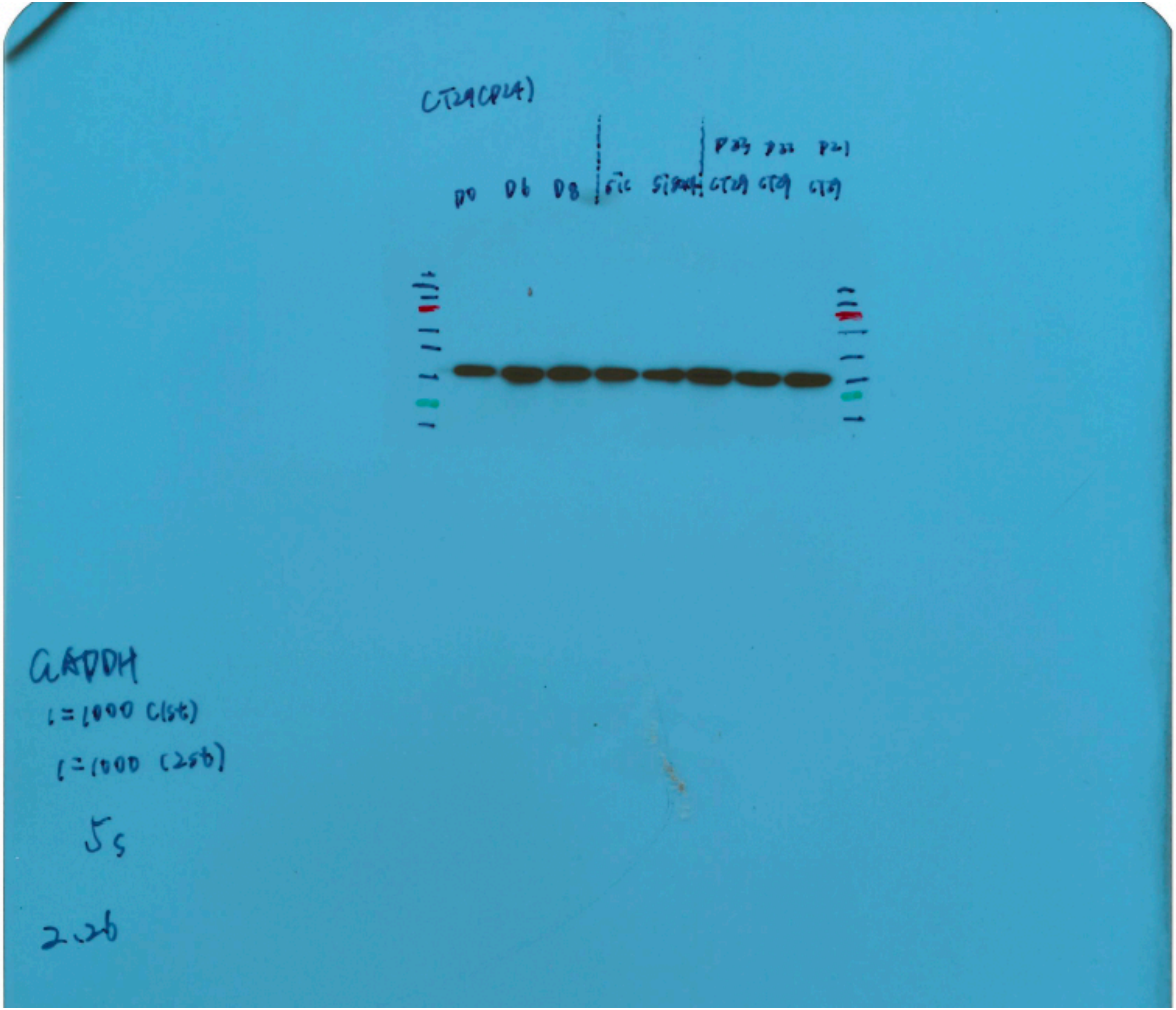
CT29(P24)
P23 P22 P21
D0 D6 D8 | SiC SiRNA | CT29 CT29 CT29
GAPDH
1:1000 (1st)
1:1000 (2nd)
5s
2.26

SOX4

1:400 (1st)

1:1000 (2nd)

30s

(60% super-signal)

2.25

CT29 (p24)

[illegible] [illegible] D01

CT29 [illegible] CT29

D0 D6 D8 siC siSox4

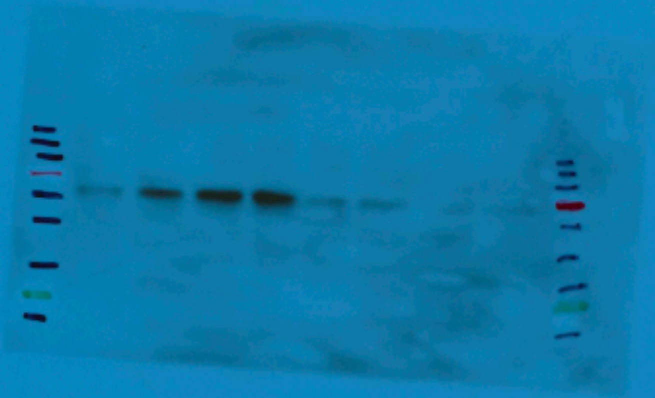

Supplement: Supplementary file 13 — Original Western Blot Image [file 41419_2026_8665_MOESM13_ESM.pdf]
